# Development and internal validation of a risk score for incident obesity among Peruvian formal workers

**DOI:** 10.1016/j.obpill.2026.100285

**Published:** 2026-06-06

**Authors:** Víctor Juan Vera-Ponce, Jhosmer Ballena-Caicedo

**Affiliations:** Facultad de Medicina (FAMED), Universidad Nacional Toribio Rodríguez de Mendoza de Amazonas (UNTRM), Amazonas, Peru

**Keywords:** (MeSH): obesity, Body mass index, Risk assessment, Prognosis, Cohort studies, Occupational health services

## Abstract

**Background:**

Adult obesity is a growing public health problem, and workplace settings offer opportunities for early identification of individuals at increased risk. Longitudinal evidence to predict the transition from normal weight or overweight to obesity among workers remains limited in Latin America. This study aimed to develop and internally validate an interpretable score to estimate incident obesity risk among Peruvian formal workers with a baseline body mass index of 18.5 to <30 kg/m^2^.

**Methods:**

This was a retrospective cohort study using occupational health records and Cox survival modeling to develop and internally validate a risk score predicting incident obesity over time among Peruvian workers without obesity at baseline. The outcome was the first subsequent assessment with BMI ≥30 kg/m^2^. Candidate Cox models were fitted using baseline clinical, behavioral, and occupational predictors. Performance was assessed using the C-index, Brier score, 36-month calibration, internal bootstrap validation with 500 successful replicates, and decision curve analysis. The score was derived from the coefficients of the final model.

**Results:**

The primary cohort included 9390 workers; 987 developed incident obesity. The final model included age, sex, baseline BMI, waist circumference, occupation, night work, smoking, alcohol use, and high baseline blood pressure. Among 7979 participants with complete data, the model had an apparent C-index of 0.857, an optimism-corrected C-index of 0.855 after 500 successful bootstrap replicates, and a 36-month IPCW Brier score of 0.091. The observed 36-month risk was 0.91%, 5.62%, and 37.05% in the low-, intermediate-, and high-risk categories, respectively.

**Conclusion:**

The score showed high discrimination and acceptable calibration for stratifying occupational risk of incident obesity. External validation and local recalibration in other occupational populations are required before routine implementation outside the source setting.

## Graphical abstract

**Figure:**
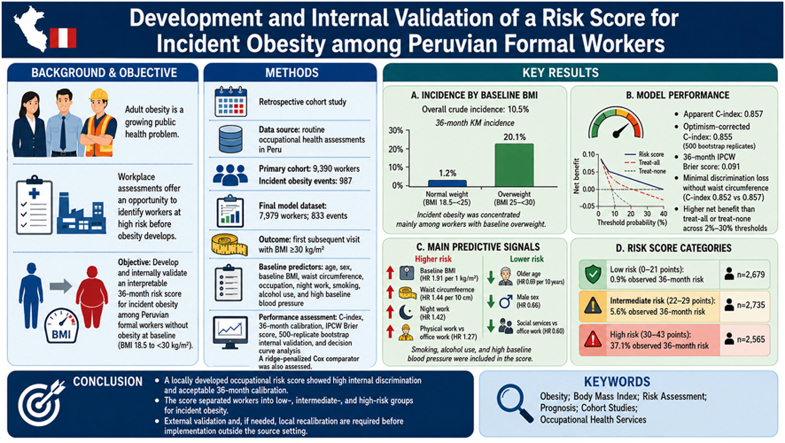

## 1 Introduction

Overweight and obesity are public health priorities because of their high frequency, persistence throughout adulthood, and association with cardiometabolic outcomes, disability, and premature mortality. In 2022, an estimated 2.5 billion adults had overweight, including more than 890 million with obesity; in the same year, 43% of adults had overweight and 16% had obesity. The global prevalence of adult obesity has more than doubled since 1990, making prevention of weight gain a central component of noncommunicable disease control strategies [1,2].

The regional burden is particularly relevant for Latin America and the Caribbean. The Region of the Americas has one of the highest prevalences of excess weight among adults, and regional reports describe that approximately one in four adults lives with obesity [3]. In Peru, the systematic review by Vasquez-Romero et al. reported that 23.23% of adults had obesity [4]. These figures position the transition from normal weight or overweight to obesity as a quantitatively important problem for preventive surveillance in adult populations.

The workplace offers a specific opportunity for early identification and prevention. Occupational health interventions focused on diet, physical activity, and environmental changes have been recommended to improve weight-related behaviors and reduce body weight, although their effects vary according to design, intensity, and implementation context. In addition, working conditions, schedules, physical demands, occupational sedentariness, and work organization may coexist with distinct anthropometric and metabolic profiles among workers. In this setting, occupational surveillance allows a shift from isolated BMI measurement to risk stratification oriented toward follow-up and preventive prioritization [5,6].

Available evidence in Peru and in the region has focused mainly on population prevalence estimates or cross-sectional associations with cardiometabolic factors [7]. In comparison, longitudinal evidence on predicting incident obesity among formal workers and on models that combine anthropometric, clinical, and occupational variables available at the start of follow-up remains limited. This gap is relevant because a useful occupational health model should not merely recognize obesity that is already present, but should identify workers without obesity who are more likely to cross the BMI ≥30 kg/m^2^ threshold during follow-up. For this information to be operational, the model must be interpretable, internally validated, and evaluated in terms of discrimination, calibration, and clinical utility.

This study aimed to develop and internally validate a prediction model to estimate the risk of incident obesity among Peruvian formal workers with normal weight or overweight at baseline. Specifically, we sought to compare candidate models based on baseline clinical and occupational predictors, select an interpretable model, derive a 36-month risk score, and evaluate its performance using discrimination, calibration, decision curve analysis, and prespecified sensitivity analyses.

## 2 Methods

### 2.1 Study design and reporting guidelines

This was a retrospective cohort study using occupational health records and Cox survival modeling to develop and internally validate a risk score predicting incident obesity over time among Peruvian workers without obesity at baseline. The data source consisted of secondary records from routine occupational medical assessments. Baseline assessment was defined as the first valid occupational visit recorded for each worker, and follow-up was reconstructed from subsequent visits with body mass index (BMI) measurement and a valid date. Reporting was structured according to TRIPOD + AI for prediction model studies developed using regression or machine learning methods (Table S1) [8].

### 2.2 Data source and study population

The database included sociodemographic, occupational, behavioral, anthropometric, hemodynamic, and, when requested by the company, biochemical information. The unit of analysis was the worker. Records were eligible if they had valid baseline BMI, a valid baseline date, and at least one subsequent dated BMI measurement. The primary cohort was restricted to workers with baseline BMI from 18.5 to <30 kg/m^2^, because the objective was to predict transition from normal weight or overweight to obesity. We excluded workers with baseline obesity, baseline underweight, missing or implausible baseline BMI, no subsequent BMI measurement, or no valid date to define follow-up.

Valid BMI was operationally defined as a value between 10 and <80 kg/m^2^. This range was used as a quality-control rule to remove obvious recording errors without imposing excessive clinical cleaning. Biochemical tests were not an inclusion criterion for the primary analysis because their availability depended on administrative decisions made by companies rather than on a homogeneous measurement protocol. Restricting the primary analysis to workers with complete laboratory data would have shifted the target population toward a subcohort partly defined by company requests for testing, creating a difficult-to-defend source of selection.

### 2.3 Baseline predictors

Candidate predictors were defined before modeling and were limited to variables available at the baseline assessment. We considered age, sex, baseline BMI, baseline waist circumference, occupation type, night work, smoking, alcohol use, and high baseline blood pressure. High baseline blood pressure was defined as systolic blood pressure ≥140 mmHg or diastolic blood pressure ≥90 mmHg. Age was scaled per 10 years, waist circumference per 10 cm, and BMI was retained as a continuous variable per kg/m^2^. Occupational categories were entered as indicator variables, with office work as the reference category.

Glucose, cholesterol, triglycerides, baseline diabetes, prediabetes, and dyslipidemia were reserved for extended models or sensitivity analyses. Final BMI, final BMI category, final obesity, and variables measured after the baseline visit were not used as predictors in the baseline model. When accumulated information during follow-up was explored, it was treated as a separate dynamic analysis and not as part of the baseline score.

### 2.4 Outcome and follow-up

The primary outcome was incident obesity, defined as the first subsequent assessment with BMI ≥30 kg/m^2^ among workers without baseline obesity. This cutoff corresponds to the usual adult BMI-based classification of obesity [2]. Because the database consisted of repeated occupational assessments rather than continuous surveillance, the outcome should be understood as obesity detected at an occupational visit, not as the exact biological onset date of obesity.

Time to event was calculated from the baseline date to the date of the first subsequent visit with BMI ≥30 kg/m^2^. Workers without an event were censored at the last subsequent visit with valid BMI <30 kg/m^2^. By design, the event was interval-censored: it was known to have occurred between the last measurement with BMI <30 kg/m^2^ and the first measurement with BMI ≥30 kg/m^2^, but the exact time at which the threshold was crossed was not observed. The detection date was used in the primary analysis because it is the operational date available for occupational surveillance. As a sensitivity analysis, the midpoint between the last visit with BMI <30 kg/m^2^ and the first visit with BMI ≥30 kg/m^2^ was used.

### 2.5 Prediction model development

The primary analysis used Cox proportional hazards models because subsequent visits were irregular and follow-up was censored. A fixed-horizon binary model would have required discarding information from workers with shorter or longer follow-up or imposing artificial time windows. The Cox model allowed use of the observed time to detection or censoring and derivation of absolute 36-month risk through the estimated baseline survival function.

Candidate models of increasing complexity were fitted. The reference model included age, sex, and baseline BMI. The minimal clinical model added waist circumference. The occupational-clinical model included age, sex, baseline BMI, baseline waist circumference, occupation type, night work, smoking, alcohol use, and high baseline blood pressure. The extended cardiometabolic model added baseline biochemical predictors and metabolic diagnoses in the subcohort with available data. An occupational-clinical model without waist circumference was also assessed to estimate the performance loss when this measurement was unavailable. The final model was selected by prioritizing discrimination, calibration, stability, completeness, and interpretability rather than isolated statistical significance.

As a prespecified penalized comparator, we fitted a ridge-penalized Cox model using the same predictor set as the final occupational-clinical model. Continuous predictors were standardized before penalization and categorical predictors were entered as indicator variables. Candidate penalty parameters (lambda = 0, 0.0001, 0.001, 0.005, 0.01, 0.05, 0.1, 0.5, 1.0, and 2.0) were evaluated using five-fold cross-validation. The tuning parameter was selected according to the highest mean cross-validated C-index. This model was used only as a predictive comparator and was not converted into the manual score.

Continuous predictors were incorporated as linear terms on the log-hazard scale to maintain parsimony and facilitate subsequent conversion to a score. This decision is debatable if markedly nonlinear relationships existed, especially for baseline BMI; therefore, calibration, subgroup performance, and the analysis restricted to baseline overweight were essential components of the evaluation. No automatic variable selection based on p values was performed.

### 2.6 Missing data

The primary analysis for each model was conducted among participants with complete information for the variables included in that model. This strategy avoided imputing unobserved information under assumptions that are difficult to verify, but it could reduce sample size and modify the analytical population. Because single median imputation of waist circumference can artificially reduce variance, the primary sensitivity analysis for missing waist circumference emphasized an occupational-clinical model that omitted waist circumference altogether. A simple sex- and baseline-BMI-category median imputation analysis was retained only as a descriptive comparison and was not used for inference or final model selection. Laboratory values were not multiply imputed because their absence was linked to company requests and could not be assumed to be missing completely at random.

### 2.7 Performance evaluation and internal validation

To improve interpretability for clinical and occupational health readers, model performance was summarized using complementary measures: discrimination to assess ranking ability, calibration to assess agreement between predicted and observed risk, and decision curve analysis to assess potential utility across risk thresholds. Discrimination was evaluated using the C-index for survival data. For the 36-month horizon, the area under the curve was also estimated among workers with known status at that horizon as a complementary analysis. Overall performance was summarized using the inverse-probability-of-censoring-weighted Brier score and the integrated Brier score between 0 and 36 months. Calibration was assessed by comparing observed and predicted 36-month risks across predicted-risk groups and using a calibration curve. These metrics were selected because discrimination alone does not assess whether absolute probabilities are numerically appropriate for risk classification [9,10].

Potential clinical utility was evaluated using 36-month decision curve analysis, comparing the net benefit of the final model against strategies of intervening in all, intervening in none, and using a reference model based on minimal predictors. This analysis was included because a model with good discrimination may not provide operational utility if it does not improve decisions within plausible risk thresholds [11].

Internal validation was performed using nonparametric bootstrap resampling to estimate C-index optimism and obtain an optimism-corrected performance measure. The final run targeted at least 500 successful bootstrap replicates; 504 resampling attempts yielded 500 successful model fits. Event sufficiency was evaluated descriptively using events per effective predictor, recognizing that simple events-per-variable rules are inferior to formal sample-size approaches for prediction models [12].

### 2.8 Score construction

The score was derived from the final Cox model. Log-HR coefficients were transformed into integer points by dividing each coefficient by a constant of 0.25 log-HR units and rounding to the nearest integer. To facilitate manual use, the total score was shifted so that the lowest observed value was zero. Absolute 36-month risk was estimated from the baseline survival function of the final model and the linear predictor corresponding to each score. Risk categories were defined after inspecting the empirical score distribution and its calibration, avoiding cutoffs fixed before performance evaluation.

### 2.9 Sensitivity analyses

Sensitivity analyses were performed to examine methodological decisions that could modify predictive estimates. First, event time was redefined using the midpoint of the detection interval. Second, fixed-horizon models at 36 and 60 months were evaluated. Third, modeling was repeated in subcohorts defined by baseline BMI category, including normal weight and overweight separately. Fourth, the model without waist circumference was emphasized as the primary sensitivity analysis for missing baseline waist data; the simple median-imputation run was retained only as a secondary descriptive comparison, and the cardiometabolic extension was evaluated in the laboratory subcohort. Fifth, early events were excluded to reduce the possibility of classifying as incident cases that were already present but undetected at baseline. In addition, alternative anthropometric plausibility rules, adjustment for calendar period, temporal validation, alternative occupational grouping, subgroup performance, approximate assessment of the proportional hazards assumption, and an exploratory landmark analysis using information accumulated before the reference horizon were evaluated.

### 2.10 Software

Analyses were performed in Python 3.13.5. We primarily used pandas and NumPy for data management, SciPy and statsmodels for auxiliary statistical procedures, lifelines for survival models, scikit-learn for metrics and predictive processing, and matplotlib for figures. The code was structured to reconstruct the analytical cohort from the source database, generate tables and figures, save individual predictions, and export the score specification.

### 2.11 Ethical considerations

The study was approved by the Ethics Committee of the Universidad Nacional Toribio Rodríguez de Mendoza de Amazonas. In addition, institutional authorization was obtained from the occupational clinic to use its database for research purposes. Data were provided in anonymized format, with all personal worker identifiers removed to ensure confidentiality. This research was conducted in accordance with the Declaration of Helsinki and standards of good research practice. Because this was a secondary analysis of data collected as part of routine occupational assessments, no additional informed consent was required. To ensure transparency and reproducibility, the anonymized database is available athttps://doi.org/10.6084/m9.figshare.27098296.v1 [13].

## 3 Results

### 3.1 Sample selection

The source database included 79,660 workers with valid baseline BMI. After excluding workers with baseline underweight, baseline obesity, or no subsequent dated BMI measurement, the primary analytical cohort comprised 9390 workers with baseline BMI between 18.5 and <30 kg/m^2^. The TRIPOD + AI checklist was structured as Table S1, and the sample selection process is shown in Fig. S1.

During follow-up, 987 dated incident obesity events were identified, defined by the first subsequent assessment with BMI ≥30 kg/m^2^. Of these events, 47 occurred among workers with normal weight at baseline and 940 among workers with overweight at baseline. Median follow-up was 25.3 months, with an interquartile range of 21.5–38.1 months. Among 62,428 workers with eligible baseline BMI and a valid baseline date, 9390 had dated subsequent follow-up, equivalent to 15.0% of the eligible baseline population. Differences between workers included and excluded because of absence of subsequent follow-up are presented in Table S2.

Workers with dated follow-up differed from those excluded because of absence of dated follow-up. Included workers were older (median age 34 vs 30 years), more frequently male (84.1% vs 77.8%), and more frequently overweight at baseline (61.6% vs 55.9%). They also had slightly higher waist circumference (median 88 vs 86 cm), whereas baseline BMI differed modestly (25.9 vs 25.5 kg/m^2^). Occupational composition was broadly similar for physical and office work, but social-service workers were more represented among those followed, whereas customer-service and health-professional categories were less represented. Because 85.0% of baseline-eligible workers lacked dated follow-up, the model should be interpreted as applying to workers with repeat occupational reassessment rather than to all workers with a single baseline examination.

### 3.2 Baseline characteristics

Baseline characteristics of the cohort are shown in Table 1. Median age was 34 years (IQR: 27 to 44), 7899 workers were men (84.1%), and 5784 had overweight at baseline (61.6%). Median baseline BMI was 25.9 kg/m^2^ (IQR: 23.8 to 27.7). Compared with workers without incident obesity, those who developed obesity during follow-up had higher median baseline BMI (28.7 vs. 25.6 kg/m^2^) and higher median baseline waist circumference (94.0 vs. 87.0 cm).

**Table 1:** Baseline characteristics of the cohort according to incident obesity.

Characteristic	Total	No incident obesity	Incident obesity
N	9390	8403	987
Age, years, median [IQR]	34 [27; 44]	34 [27; 44]	35 [27; 43]
Baseline BMI, kg/m^2^, median [IQR]	25.9 [23.8; 27.7]	25.6 [23.5; 27.3]	28.7 [27.7; 29.4]
Baseline waist circumference, cm, median [IQR]	88 [82; 94]	87 [82; 93]	94 [90; 98.8]
Baseline SBP, mmHg, median [IQR]	113 [103; 118]	113 [103; 113]	113 [103; 123]
Baseline DBP, mmHg, median [IQR]	73 [63; 73]	73 [63; 73]	73 [63; 83]
Baseline glucose, mg/dL, median [IQR]	91 [83; 97]	91 [83; 97]	91 [82; 98]
Baseline cholesterol, mg/dL, median [IQR]	188 [164; 214]	188 [164; 214]	190 [170; 215]
Baseline triglycerides, mg/dL, median [IQR]	116 [85; 162]	115 [85; 160]	129 [92; 172]
Sex			
Male	7899 (84.1)	7056 (84)	843 (85.4)
Female	1491 (15.9)	1347 (16)	144 (14.6)
Baseline BMI category			
Normal weight	3606 (38.4)	3559 (42.4)	47 (4.8)
Overweight	5784 (61.6)	4844 (57.6)	940 (95.2)
Occupation type			
Customer service	66 (0.7)	61 (0.7)	5 (0.5)
Health professionals	142 (1.5)	126 (1.5)	16 (1.6)
Social services	565 (6)	519 (6.2)	46 (4.7)
Physical work	5173 (55.1)	4593 (54.7)	580 (58.8)
Office work	3444 (36.7)	3104 (36.9)	340 (34.4)
Night work			
No	8837 (94.1)	7960 (94.7)	877 (88.9)
Yes	553 (5.9)	443 (5.3)	110 (11.1)
Smoking			
No	5653 (60.2)	5086 (60.5)	567 (57.4)
Yes	3737 (39.8)	3317 (39.5)	420 (42.6)
Alcohol use			
No	4677 (49.8)	4185 (49.8)	492 (49.8)
Yes	4713 (50.2)	4218 (50.2)	495 (50.2)
High baseline blood pressure			
No	9092 (97)	8136 (97)	956 (97)
Yes	284 (3)	254 (3)	30 (3)
Baseline diabetes			
No	7764 (98.1)	6929 (98.1)	835 (98.5)
Yes	148 (1.9)	135 (1.9)	13 (1.5)
Baseline prediabetes			
No	6751 (87)	6043 (87.2)	708 (84.8)
Yes	1013 (13)	886 (12.8)	127 (15.2)

Night work was present in 553 workers (5.9%) and was more frequent among those who developed incident obesity (11.1%) than among those who did not (5.3%). Baseline smoking was recorded in 3737 workers (39.8%) and alcohol use in 4713 (50.2%). High baseline blood pressure was infrequent, with 284 cases (3.0%). Completeness was 100% for age, sex, baseline BMI, occupation, night work, smoking, and alcohol use. Baseline waist circumference had 1400 missing values (14.9%); glucose, cholesterol, and triglycerides had 1478 (15.7%), 4726 (50.3%), and 4699 (50.0%) missing values, respectively. The missing-data pattern is summarized in Table S3.

### 3.3 Incidence of incident obesity

**Fig. 1:**
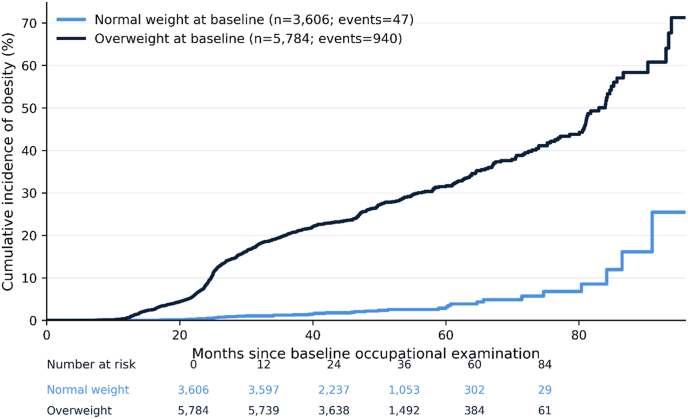
Cumulative incidence of incident obesity according to baseline BMI category.

The crude incidence of incident obesity was 10.5% in the primary cohort. Crude incidence was 1.3% among workers with normal weight at baseline and 16.3% among workers with overweight at baseline. At 36 months, Kaplan-Meier-estimated cumulative incidence was 1.2% in baseline normal weight and 20.1% in baseline overweight. Cumulative incidence curves by baseline BMI category are presented in Fig. 1, and the number at risk, events, and censoring by follow-up interval are shown in Table S4.

In subgroup analyses, 36-month cumulative incidence was 13.3% in men and 12.1% in women. By baseline age, 36-month cumulative incidence was 12.4% among workers younger than 30 years, 14.7% in those aged 30–39 years, 13.0% in those aged 40–49 years, and 11.7% in those aged 50 years or older. By occupation, 36-month cumulative incidence was 14.9% in physical work, 11.2% in office work, 10.9% in social services, 16.9% in health professionals, and 4.9% in customer service. Among workers with night work, 36-month cumulative incidence was 19.4%, compared with 12.7% among those without night work. These results are presented in Table 2.

**Table 2:** Incidence of incident obesity according to baseline subgroups.

Characteristic	n	Events	Crude risk (%)	36-month KM incidence (%)	Median follow-up (months)
**Baseline BMI category**					
Normal weight	3606	47	1.3	1.2	25.6
Overweight	5784	940	16.3	20.1	25.3
**Sex**					
Male	7899	843	10.7	13.3	25.4
Female	1491	144	9.7	12.1	25.1
**Age**					
30–39	2873	335	11.7	14.7	25.6
40–49	1915	200	10.4	13.0	25.3
<30	3169	314	9.9	12.4	24.9
≥50	1433	138	9.6	11.7	25.9
**Occupation**					
Customer service	66	5	7.6	4.9	25.3
Health professionals	142	16	11.3	16.9	24.4
Social services	565	46	8.1	10.9	29.7
Physical work	5173	580	11.2	14.9	25.0
Office work	3444	340	9.9	11.2	25.6
**Night work**					
No	8837	877	9.9	12.7	25.3
Yes	553	110	19.9	19.4	25.8

### 3.4 Development and comparison of prediction models

Operational definitions of variables are presented in Table S5, and the specification of candidate models is presented in Table S6. The reference model included age, sex, and baseline BMI; the minimal clinical model added waist circumference; the occupational-clinical model additionally incorporated occupation, night work, smoking, alcohol use, and high baseline blood pressure; and the extended cardiometabolic model added available laboratory variables. Full coefficients for candidate models are presented in Table S7.

The reference model had an apparent C-index of 0.849 and a 36-month IPCW Brier score of 0.086. The minimal clinical model had an apparent C-index of 0.852 and a 36-month IPCW Brier score of 0.092. The occupational-clinical model with waist circumference, selected as the final model, was estimated in 7979 workers and 833 events, with 12 effective predictors, 69.4 events per effective predictor, an apparent C-index of 0.857, a 36-month IPCW Brier score of 0.091, and an integrated Brier score of 0.041. The extended laboratory model had an apparent C-index of 0.864 and a 36-month IPCW Brier score of 0.074, but it was estimated in a subcohort of 3219 workers and 311 events. The model without waist circumference was estimated in 9376 workers and 986 events, with an apparent C-index of 0.852 and a 36-month IPCW Brier score of 0.086. Performance comparison is presented in Table 3.

**Table 3:** Candidate prediction models and comparative performance.

Model	Description	n	Events	Effective predictors	Events per predictor	Apparent C-index	36-month IPCW Brier	0-36-month IPCW IBS	n with known 36-month status	36-month AUC among those with known status	36-month Brier among those with known status	Optimism-corrected C-index
M0_reference	Age + sex + baseline BMI	9390	987	3	329	0.85	0,086	0.04	3308	0,868	0,127	
M1_minimal_clinical	Age + sex + baseline BMI + waist circumference	7990	834	4	208	0.85	0,092	0.04	2527	0,873	0,138	
M2_occupational_clinical	Occupational-clinical model with waist circumference	7979	833	12	69.4	0.86	0,091	0.04	2522	0,878	0,135	0,855
M3_extended_laboratory	Extended laboratory model	3219	311	16	19.4	0.86	0,074	0.03	1159	0,893	0,111	
M4_without_waist_circumference	Occupational-clinical model without waist circumference	9376	986	11	89.6	0.85	0,086	0.04	3303	0,871	0,126	

The ridge-penalized Cox comparator used the same predictor set as the final occupational-clinical model. Five-fold cross-validation selected lambda = 0.005. This model had an apparent C-index of 0.857 and a mean cross-validated C-index of 0.853 (SD 0.016). Its discrimination was essentially identical to that of the interpretable Cox model, so it did not justify replacing the manually applicable score.

### 3.5 Final model and score

In the final occupational-clinical model with waist circumference, baseline BMI was associated with a higher hazard of incident obesity (HR per 1 kg/m^2^: 1.91; 95% CI: 1.81 to 2.02). Baseline waist circumference also showed a positive association (HR per 10 cm: 1.44; 95% CI: 1.26 to 1.65). Baseline age per 10 years had an HR of 0.69 (95% CI: 0.65 to 0.74), and male sex had an HR of 0.66 (95% CI: 0.54 to 0.81). Night work had an HR of 1.42 (95% CI: 1.14 to 1.77). Compared with office work, physical work had an HR of 1.27 (95% CI: 1.10 to 1.48), and social services had an HR of 0.60 (95% CI: 0.43 to 0.82). Smoking, alcohol use, and high baseline blood pressure had confidence intervals that included the null. Coefficients, HRs, confidence intervals, p values, and assigned points are shown in Table 4.

**Table 4:** Final model with coefficients, HRs, and score-point assignment.

Predictor	Model variable	Log-HR coefficient	SE	HR	95% CI HR	p	Score points	Point note
Baseline age (per 10 years)	age10_c	−0,365	0,036	0,694	0.65–0.74	<0,001	−1	1 point ≈ 0.25 log-HR
Baseline BMI (per 1 kg/m^2^)	baseline_bmi_c	0,647	0,029	1909	1.81–2.02	<0,001	3	1 point ≈ 0.25 log-HR
Baseline waist circumference (per 10 cm)	waist_circumference10_c	0,364	0,070	1439	1.26–1.65	<0,001	1	1 point ≈ 0.25 log-HR
Male sex	male_sex	−0,421	0,104	0,657	0.54–0.81	<0,001	−2	1 point ≈ 0.25 log-HR
Night work	night_work	0,349	0,112	1418	1.14–1.77	0,002	1	1 point ≈ 0.25 log-HR
Baseline smoking	smoking	−0,038	0,071	0,963	0.84–1.11	0,596	0	1 point ≈ 0.25 log-HR
Baseline alcohol use	alcohol	0,041	0,070	1042	0.91–1.19	0,558	0	1 point ≈ 0.25 log-HR
High baseline blood pressure	high_baseline_bp	−0,244	0,232	0,783	0.5–1.23	0,292	−1	1 point ≈ 0.25 log-HR
Occupation: physical work vs office work	occupation_Physical_work	0,242	0,077	1274	1.1–1.48	0,002	1	1 point ≈ 0.25 log-HR
Occupation: customer service vs office work	occupation_Customer_service	−0,128	0,581	0,880	0.28–2.75	0,825	−1	1 point ≈ 0.25 log-HR
Occupation: health professionals vs office work	occupation_Health_professionals	0,235	0,284	1265	0.72–2.21	0,408	1	1 point ≈ 0.25 log-HR
Occupation: social services vs office work	occupation_Social_services	−0,516	0,165	0,597	0.43–0.82	0,002	−2	1 point ≈ 0.25 log-HR

The score was derived from the log-HR coefficients of the final model, using an approximate conversion of 1 point for each 0.25 log-HR units. Score categories were low risk (0–21 points), intermediate risk (22–29 points), and high risk (30–43 points). The low-risk category included 2679 workers (33.6%), with 23 events and an observed 36-month risk of 0.9%. The intermediate category included 2735 workers (34.3%), with 109 events and an observed 36-month risk of 5.6%. The high-risk category included 2565 workers (32.1%), with 701 events and an observed 36-month risk of 37.1%. Categories and observed and predicted risks are presented in Table 5. The full point-to-absolute-risk conversion and tabular score calculator are presented in Tables S11 and S12.

**Table 5:** Score categories and estimated absolute 36-month risk.

Score category	Point range	n	Proportion (%)	Events	Mean predicted 36-month risk (%)	Observed KM 36-month incidence (%)	36-month O/E
Low	0–21	2679	33.6	23	0.9	0.9	0.99
Intermediate	22–29	2735	34.3	109	6.7	5.6	0.84
High	30–43	2565	32.1	701	35.0	37.1	1.06

### 3.6 Internal validation, calibration, and clinical utility

Internal bootstrap validation of the final model included 500 successful replicates after 504 attempted resamples. Mean C-index optimism was 0.00184, and the optimism-corrected C-index was 0.8547. The percentile interval for the optimism-corrected C-index ranged from 0.8424 to 0.8678. Detailed internal validation results are presented in Table S8 and S8b.

**Fig. 2:**
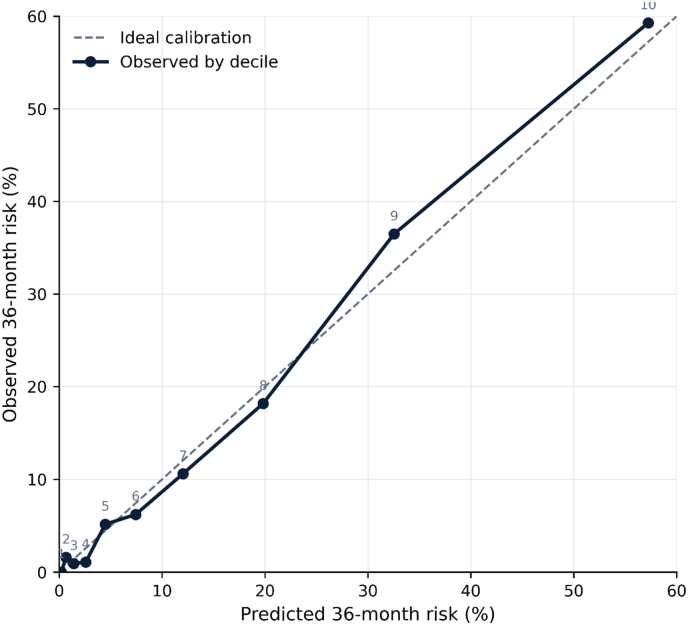
Calibration of the final model at 36 months.

Calibration at 36 months was evaluated using the observed-versus-predicted curve for the final model, presented in Fig. 2. Across deciles of predicted risk, observed risk increased progressively from the lower deciles to the highest decile. In the highest-risk decile, mean predicted risk was 57.2%, and observed 36-month incidence was 59.3%. In the ninth decile, mean predicted risk was 32.5%, and observed incidence was 36.5%. Tabular calibration by deciles is presented in Table S9.

**Fig. 3:**
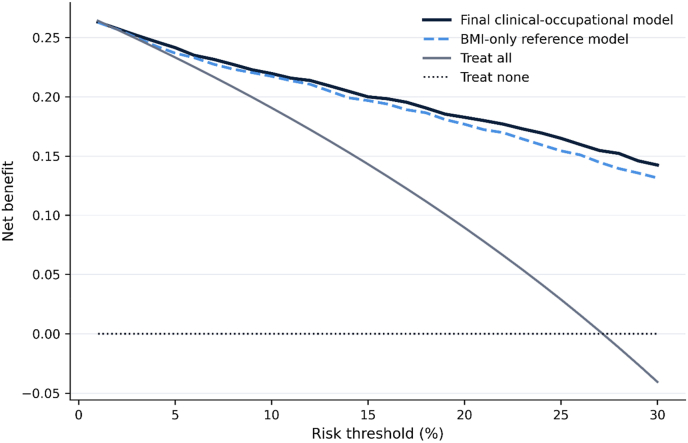
Clinical decision curve of the final model at 36 months.

Clinical utility was evaluated using 36-month decision curve analysis. Fig. 3 shows the net benefit of the final model compared with the BMI-based reference model, the treat-all strategy, and the treat-none strategy. Across risk thresholds from 2% to 30%, the final model showed higher net benefit than treating all or treating none. Compared with the baseline BMI reference model, the net benefit increase was small and consistent across the evaluated range. Numerical net-benefit values by threshold are presented in Table S10.

### 3.7 Sensitivity analyses

When the event date was redefined as the midpoint between the last visit with BMI <30 kg/m^2^ and the first visit with BMI ≥30 kg/m^2^, the occupational-clinical model maintained an apparent C-index of 0.856 and a 36-month AUC of 0.884 among workers with known status at that horizon. These results are presented in Table S13.

In fixed-horizon analyses, among 2522 workers with known 36-month status, 685 events were identified and the AUC of the risk derived from the Cox model was 0.878. At 60 months, among 1065 workers with known status, 803 events were identified and the AUC was 0.880. The Brier score was 0.135 at 36 months and 0.198 at 60 months, as shown in Table S14.

In the baseline normal-weight subcohort, there were 47 events and the separate model could not be estimated because of matrix singularity. In the baseline overweight subcohort, the occupational-clinical model with waist circumference was estimated in 4902 workers and 794 events, with an apparent C-index of 0.799, a 36-month IPCW Brier score of 0.135, and an integrated Brier score of 0.062. Subcohort analyses are presented in Table S15.

The model without waist circumference included 9376 workers and 986 events, with an apparent C-index of 0.852 and a 36-month IPCW Brier score of 0.086. Compared with the final model with waist circumference, the change in discrimination was small (0.852 vs 0.857). The median-imputed waist-circumference analysis showed an apparent C-index of 0.856 and a 36-month IPCW Brier score of 0.085, but this result was treated only as descriptive because single median imputation can artificially reduce variance. These results are shown in Table S16. The extended laboratory model is presented in Table S17.

Exclusion of events detected before 6 months yielded 832 events and an apparent C-index of 0.856. Exclusion of events before 12 months yielded 804 events and an apparent C-index of 0.856. The strict definition with subsequent confirmation reduced confirmed events to 133 and events used in the model to 109. These analyses are presented in Table S18.

Alternative rules for plausible BMI values did not change the cohort size or the number of events: the restrictions 10 to <80, 12 to <70, and 15 to <60 kg/m^2^ maintained 9390 workers and 987 events. The distribution by baseline year showed longer median follow-up in earlier years and shorter follow-up in recent periods; for example, median follow-up was 64.6 months for 2013 and 15.3 months for 2020. These results are presented in Tables S19 and S20.

In temporal validation, using a cutoff on 2017-08-26, the development set included 3993 workers and 381 events, whereas the validation set included 3986 workers and 452 events. The validation C-index was 0.838, and the 36-month Brier score was 0.173. The proportion with dated subsequent follow-up among baseline eligible workers with a date was 15.0%, as detailed in Table S22. Temporal validation is presented in Table S21.

The occupational grouping used in the model included physical work, office work, social services, health professionals, and customer service, with frequencies described in Table S23. Proportionality diagnostics based on correlations of residuals with log-time did not show high absolute correlations; correlations ranged from −0.111 to 0.078 for the evaluated predictors, according to Table S24.

Subgroup performance showed a C-index of 0.852 in men and 0.879 in women. By baseline BMI category, the C-index was 0.696 in normal weight and 0.799 in overweight. Among workers with night work, the C-index was 0.796, and the mean predicted 36-month risk was 22.5%, compared with an observed incidence of 23.1%. Complete subgroup results are presented in Table S25. The exploratory landmark analysis included 1456 workers, with 111 future events and a median time to landmark of 12.4 months; this analysis is summarized in Table S26.

## 4 Discussion

### 4.1 Main findings

In this retrospective cohort of Peruvian formal workers with normal weight or overweight at baseline, incident obesity developed frequently during follow-up and was concentrated mainly among those who started with overweight. The occupational-clinical model with age, sex, baseline BMI, waist circumference, occupation, night work, smoking, alcohol use, and high baseline blood pressure showed high internal discrimination, with a bootstrap-corrected C-index of 0.855 after 500 successful bootstrap replicates, and allowed derivation of a manually applicable score. Score stratification separated groups with observed 36-month risks of 0.91%, 5.62%, and 37.05% in the low-, intermediate-, and high-risk categories, respectively. These results indicate that baseline information available in occupational health assessments can differentially classify the risk of transition to obesity without incorporating final variables or post-baseline measurements.

### 4.2 Comparison with other studies

The observed performance should be interpreted in relation to previous models for predicting obesity or weight gain. OPoRT, developed in Canada to estimate population-level 10-year obesity risk using longitudinal surveys, reported a C-statistic ≥0.89 and acceptable calibration using survey variables, including baseline BMI, age, smoking, alcohol use, physical activity, and sociodemographic characteristics [14]. By contrast, the European EPIC score for substantial 5-year weight gain had more limited discrimination, with an AUC of 0.64 in derivation and 0.57 in external validation [15]. The performance of the present model is closer to OPoRT than to the EPIC score; however, the comparison is not direct because this study predicted crossing to BMI ≥30 kg/m^2^ among workers without obesity at baseline, whereas EPIC predicted ≥10% weight gain from baseline in a more heterogeneous general population. Part of the high discrimination of the current model likely derives from baseline BMI placing some workers close to the diagnostic threshold for obesity.

The relevance of baseline BMI and waist circumference is also consistent with studies showing that the definition and measurement of adiposity substantially modify risk classification. In Peru, a systematic review found that obesity prevalence varies widely depending on whether BMI, waist circumference, or waist-to-height ratio is used, reinforcing that central anthropometry provides information that is not identical to BMI [4]. Similarly, a study using machine-learning algorithms in adults with overweight identified waist circumference, hip circumference, sex, and systolic blood pressure among the main predictors of obesity, although its design was cross-sectional and therefore not comparable as longitudinal incidence prediction [16]. In this study, waist circumference added predictive information to baseline BMI, but the model without waist circumference retained similar performance, suggesting that its utility should be weighed against availability, measurement quality, and operational implementation.

The occupational findings are compatible with the literature on shift work and excess weight, although they should not be read as causal estimates. A meta-analysis of observational studies reported an association between shift work and higher risk of overweight or obesity, with RRs of 1.25 for overweight and 1.17 for obesity; the pattern was also heterogeneous according to shift type and study design [17]. Another meta-analysis focused on specific obesity types also reported an association between night work and excess weight, with evidence of a gradient according to exposure characteristics in some studies [18]. In the present cohort, baseline night work was associated with a higher hazard of incident obesity within the prediction model, but the database did not allow differentiation by fixed versus rotating shifts, cumulative duration, chronotype, sleep, nocturnal eating, or physical activity. Therefore, night work functions here as an occupational predictive marker and not as evidence of a specific etiologic mechanism.

Comparison with recent machine-learning models requires methodological caution. Some machine-learning studies for obesity classification have reported high AUCs, such as the CatBoost model in adults with overweight, with a test AUC of 0.87 [16]. However, several of these studies are cross-sectional, use variables closely related to the outcome definition, or do not prioritize calibration, temporal validation, and clinical utility. In contrast, this study used a longitudinal outcome, separated baseline model development from any subsequent information, and evaluated calibration, Brier score, decision curve analysis, and internal validation according to principles for prognostic model evaluation and prediction model reporting [[8], [9], [10]]. Choosing an interpretable Cox model over more complex models is consistent with the goal of producing a score applicable to occupational surveillance, not an opaque classification exercise.

### 4.3 Public health and international implications

The first implication is that the study focuses on a preventable phase in the natural history of excess weight: the transition from normal weight or overweight to obesity. Globally, the prevalence of adult overweight and obesity has increased steadily, and Global Burden of Disease projections anticipate a growing burden toward 2050 [1]. This pattern overlaps with the burden documented in the Americas and with Peruvian national estimates of excess weight in adults [1,3]. A model that identifies high-risk workers before they cross the obesity threshold may help shift the response from late detection toward selective prevention, provided it is linked to feasible and evaluable interventions.

The second implication is operational. Occupational assessments generate periodic measurements of weight, height, waist circumference, blood pressure, and health behaviors, but these data are often used only for fitness-for-work decisions or administrative reporting. This study shows that such records can become a platform for cardiometabolic risk stratification. Reviews of workplace interventions have shown modest reductions in weight or BMI when nutritional components, physical activity, and environmental or multicomponent strategies are combined [19,20].

In that context, a score can help prioritize the intensity and frequency of counseling, anthropometric follow-up, and preventive referral, rather than applying homogeneous interventions to workers with markedly different risks.

The third implication is its potential transferability to occupational health systems in middle-income countries. The primary model does not depend on laboratory tests, imaging, or costly measurements, increasing its applicability in contexts where biochemical test availability depends on company decisions rather than uniform clinical protocols. This characteristic differentiates it from models that are more dependent on extensive or high-granularity data and brings it closer to population tools such as OPoRT, which were designed to operate using routinely collected information [14]. Before programmatic use outside the original database, the score requires external validation, assessment of local recalibration, and impact analysis, in line with current standards for prediction models [8].

### 4.4 Limitations

This study has limitations. First, it was based on secondary occupational records, with irregular visits and an interval-censored outcome; the date used corresponds to obesity detection and not necessarily to the biological time of onset. Second, only 15.0% of baseline-eligible workers had dated subsequent follow-up, so selection by employment continuity, examination periodicity, or company requirements may affect transportability to all workers assessed only once. Third, laboratory data had substantial and probably nonrandom missingness and therefore were not part of the primary model. Fourth, diet, physical activity, sleep, chronotype, stress, income, education, and cumulative duration of night work were unavailable, although these variables are relevant for explaining risk heterogeneity. Fifth, the outcome used BMI ≥30 kg/m^2^; although this is a standard definition, BMI does not distinguish fat mass from lean mass or adipose distribution, and in Peru obesity estimates may vary according to the anthropometric indicator used [4]. Sixth, although predictors were measured at baseline, reverse causality cannot be ruled out or, more precisely, some predictors may reflect metabolic or behavioral processes that preceded the diagnosis of obesity detected during follow-up. This limitation affects any etiologic interpretation; the analysis was predictive and did not aim to estimate causal effects. Seventh, validation was internal and based on bootstrap resampling; therefore, external validation is required before routine clinical or occupational use.

## 5 Conclusion

Among Peruvian formal workers with normal weight or overweight at baseline, it was possible to develop an interpretable prediction model for incident obesity using baseline clinical and occupational variables. The final model showed high internal discrimination, acceptable 36-month calibration, and potential utility for separating risk groups. The derived score identifies a high-risk category with substantially higher cumulative incidence than the low- and intermediate-risk categories, supporting its further evaluation as a preventive surveillance tool in occupational health. External validation, local recalibration, and implementation studies are still required before routine use outside the source occupational health system.

### 5.1 Takeaway messages

• A baseline clinical-occupational Cox score stratified 36-month incident obesity risk among Peruvian formal workers without obesity at baseline.
• Omitting waist circumference produced only a small decrease in discrimination, supporting a simpler implementation where waist measurement is unavailable, although calibration should be locally checked.
• External validation, local recalibration, and impact studies are required before routine implementation in other occupational populations.

## Ethics approval and consent to participate

This study used previously collected secondary data and was approved by the Research Ethics Committee of the Universidad Nacional Toribio Rodríguez de Mendoza de Amazonas. In addition, because the analysis was based exclusively on anonymized/de-identified data from an existing database, no additional informed consent was required for this study, in accordance with national regulations and applicable ethical guidelines.

## Author contributions

**Víctor Juan Vera-Ponce:** Conceptualization, Investigation, Formal analysis, Methodology, Software, Funding acquisition, Supervision, Resources, Visualization, Writing - original draft, Writing - review and editing.

**Jhosmer Ballena-Caicedo:** Conceptualization, Investigation, Methodology, Project administration, Validation, Data curation, Writing - original draft, Writing - review and editing.

## Declaration on the use of artificial intelligence in writing

The authors declare that generative artificial intelligence (AI) or large language models (LLMs) were not used in the conception, data analysis, or initial drafting of this manuscript.

## Funding

Article processing charges, if applicable upon acceptance, will be covered by the funding source (Vicerrectorado de Investigación de la Universidad Nacional Toribio Rodríguez de Mendoza de Amazonas), which had no role in study design, data collection and analysis, decision to publish, or preparation of the manuscript.

## Funding

This study was funded by the Vice-Rectorate for Research of the Universidad Nacional Toribio Rodríguez de Mendoza de Amazonas.

## Data Availability

The data supporting the findings of this study are available at the following link: 10.6084/m9.figshare.27098296. The code can be shared upon request.
