# Supplementary material for: Development and internal validation of a risk score for incident obesity among Peruvian formal workers
Source: Obes Pillars. 2026 Jun 6;19:100285. doi: 10.1016/j.obpill.2026.100285 (PMC13264318; doi:10.1016/j.obpill.2026.100285)

**SUPPLEMENTARY TABLES**

Table S1. TRIPOD+AI checklist

| **Item** | **Topic** | **Reporting recommendation** | **Reported** | **Location in the manuscript** |
| --- | --- | --- | --- | --- |
| **TITLE** | | | | |
| **1** | **Title** | Identify the study as development or performance evaluation of a multivariable prediction model, the target population, and the outcome to be predicted. | **Yes** | Main title |
| **ABSTRACT** | | | | |
| **2** | **Abstract** | Report a structured abstract according to the TRIPOD+AI for Abstracts checklist (13 items). | **Yes** | Structured abstract |
| **INTRODUCTION** | | | | |
| **3a** | **Background and rationale** | Explain the health context (diagnostic/prognostic) and the rationale for developing or evaluating the model, with reference to existing models. | **Yes** | Introduction, paragraphs 1-4 |
| **3b** | **Target population and purpose** | Describe the target population and intended purpose of the model in the care pathway, including its intended users. | **Partial** | Introduction, paragraph 4 |
| **3c** | **Health inequities** | Describe any known health inequities across sociodemographic groups in the target population. | **No** | Absent from the Introduction |
| **4** | **Objectives** | Specify the study objectives, including whether it describes development, validation, or both. | **Yes** | Introduction, final paragraph |
| **METHODS** | | | | |
| **5a** | **Data: sources** | Describe the data sources separately for the development and evaluation sets, their justification, and representativeness. | **Yes** | Methods, Data source and study population |
| **5b** | **Data: dates** | Specify the dates of the data, including the start and end of recruitment and, where applicable, the end of follow-up. | **Yes** | Results, temporal validation and Table S20 |
| **6a** | **Participants: setting** | Specify key elements of the setting (primary care, secondary care, general population), and the number and location of centers. | **Partial** | Methods |
| **6b** | **Participants: eligibility criteria** | Describe participant eligibility criteria. | **Yes** | Methods, Data source and study population |
| **6c** | **Participants: treatments** | Detail treatments received and how they were handled in development or validation, if relevant. | **No** | Not reported |
| **7** | **Data preparation** | Describe preprocessing and quality controls, including whether they were similar across sociodemographic groups. | **Partial** | Methods, Baseline predictors |
| **8a** | **Outcome: definition** | Define the outcome and time horizon, how and when it was assessed, and the rationale for its choice. | **Yes** | Methods, Outcome and follow-up |
| **8b** | **Outcome: subjective interpretation** | If outcome assessment requires subjective interpretation, describe the qualifications and characteristics of the assessors. | **N/A** | — |
| **8c** | **Outcome: blinding** | Report actions taken to assess the outcome blinded to predictors. | **Partial** | Not explicitly reported |
| **9a** | **Predictors: initial selection** | Describe the choice of initial predictors (literature, previous models, all available variables) and any preselection before modeling. | **Yes** | Methods, Baseline predictors |
| **9b** | **Predictors: definition** | Define all predictors, how and when they were measured, and any blinding procedures. | **Yes** | Methods, Baseline predictors and Table S5 |
| **9c** | **Predictors: subjective interpretation** | If measurement requires subjective interpretation, describe the qualifications and characteristics of the assessors. | **N/A** | — |
| **10** | **Sample size** | Explain how the sample size was determined and justify its adequacy, separately for development and evaluation. | **Yes** | Methods, Performance evaluation and internal validation; Results, Prediction models |
| **11** | **Missing data** | Describe how missing data were handled and the reasons for any omission. | **Yes** | Methods, Missing data and Table S3 |
| **12a** | **Analytical methods: use of data** | Describe how the data were used (development, evaluation), whether they were partitioned, considering sample-size requirements. | **Yes** | Methods, Model development and Sensitivity analyses |
| **12b** | **Analytical methods: handling of predictors** | Describe handling of predictors in the analysis (functional form, scaling, transformation, standardization). | **Yes** | Methods, Prediction model development |
| **12c** | **Analytical methods: model type and internal validation** | Specify the model type, rationale, all construction steps including hyperparameter tuning, and the method of internal validation. | **Yes** | Methods, Prediction model development |
| **12d** | **Analytical methods: heterogeneity/clustering** | Describe whether and how heterogeneity in parameters and performance across clusters (hospitals, countries) was handled. | **No** | Absent |
| **12e** | **Analytical methods: performance measures** | Specify all measures and plots used to evaluate performance (discrimination, calibration, clinical utility). | **Yes** | Methods, Performance evaluation and internal validation |
| **12f** | **Analytical methods: model updating** | Describe model updating (recalibration) after evaluation, overall or by subgroups. | **N/A** | — |
| **12g** | **Analytical methods: calculating predictions** | For evaluation, describe how predictions were calculated (formula, code, object, API). | **Yes** | Methods, Score construction; Tables 4, S11, S12 |
| **13** | **Class imbalance** | If methods for class imbalance were used, state why and how, and any subsequent recalibration. | **N/A** | — |
| **14** | **Fairness** | Describe approaches used to address model fairness and their justification. | **No** | Absent |
| **15** | **Model output** | Specify the model output (probabilities, classification), rationale, and thresholds. | **Yes** | Methods, Score construction; Results, Final model and score |
| **16** | **Development vs evaluation: differences** | Identify differences between the development and evaluation data in setting, criteria, outcome, and predictors. | **N/A** | — |
| **17** | **Ethical approval** | Name the committee that approved the study and describe consent or the waiver. | **Yes** | Yes. The manuscript states that the study was approved by an ethics committee |
| **OPEN SCIENCE** | | | | |
| **18a** | **Funding** | State the funding source and the role of funders in the study. | **Yes** | The manuscript states that the study was self-funded |
| **18b** | **Conflicts of interest** | Declare conflicts of interest and financial disclosures for all authors. | **Yes** | The manuscript states that there are no conflicts of interest |
| **18c** | **Protocol** | State where the study protocol can be accessed or declare that none was prepared. | **Yes** | The project has not been registered in a registry but was reviewed by an ethics committee |
| **18d** | **Registration** | Provide study registration information or declare that the study was not registered. | **Yes** | The project has not been registered in a registry but was reviewed by an ethics committee |
| **18e** | **Data sharing** | Provide details on the availability of study data. | **Yes** | The text indicates where the data can be accessed |
| **18f** | **Code sharing** | Provide details on the availability of analytical code. | **Yes** | The code may be shared upon request |
| **PATIENT AND PUBLIC INVOLVEMENT** | | | | |
| **19** | **Patient and public involvement** | Detail patient and public involvement in design, conduct, reporting, interpretation, or dissemination, or declare its absence. | **Yes** | The study was a data analysis |
| **RESULTS** | | | | |
| **20a** | **Participants: flow** | Describe participant flow throughout the study, with numbers with/without the outcome and a summary of follow-up. Flow diagram. | **Yes** | Results, Sample selection; Figure S1 |
| **20b** | **Participants: characteristics** | Report characteristics overall and by source/setting, including key dates, predictors, treatments, sample size, events, follow-up, and missingness. | **Yes** | Results, Baseline characteristics; Table 1 |
| **20c** | **Comparison of development vs evaluation data** | For evaluation, show comparison with the development data in the distribution of important predictors. | **N/A** | — |
| **21** | **Model development: sample sizes** | Specify the number of participants and events in each analysis (development, tuning, evaluation). | **Yes** | Results, Model development and comparison; Table 3 |
| **22** | **Model specification** | Provide the final prediction model in sufficient detail to allow its use (coefficients, intercept, baseline survival function, equations). | **Yes** | Results, Final model and score; Table 4; Tables S11-S12 |
| **23** | **Model performance** | Report model performance: discrimination, calibration (graphical and numerical), and, where relevant, clinical utility with corresponding CIs. | **Partial** | Results, Internal validation, calibration, and clinical utility; Figures 2-3 |
| **24** | **Model updating** | Report results of model updating (recalibration) overall or by group. | **N/A** | — |
| **DISCUSSION** | | | | |
| **25** | **Interpretation** | Provide an overall interpretation of results, considering the objectives, limitations, findings from similar studies, and other relevant evidence. | **Yes** | Discussion, Main findings and Comparison with other studies |
| **26** | **Limitations** | Discuss study limitations (potential sources of bias or imprecision) and implications for model use. | **Yes** | Discussion, Limitations |
| **27** | **Usability of the model in the current care context** | Describe how the model is expected to be used in practice, including implementation-relevant aspects and potential barriers. | **Partial** | Discussion, Public health and international implications |

Table S2. Baseline characteristics of workers included and excluded because of absence of dated follow-up.

| Characteristic | Group | Value | n |
| --- | --- | --- | --- |
| Age, median [IQR] | Excluded without dated follow-up | 30 [24; 38] | 53038 |
| Age, median [IQR] | Included with dated follow-up | 34 [27; 44] | 9390 |
| BMI baseline, median [IQR] | Excluded without dated follow-up | 25.5 [23.3; 27.5] | 53038 |
| BMI baseline, median [IQR] | Included with dated follow-up | 25.9 [23.8; 27.7] | 9390 |
| Waist circumference, median [IQR] | Excluded without dated follow-up | 86 [80; 92] | 53038 |
| Waist circumference, median [IQR] | Included with dated follow-up | 88 [82; 94] | 9390 |
| Sex |  |  |  |
| Male | Excluded without dated follow-up | 41280 (77.8) | 53038 |
| Female | Excluded without dated follow-up | 11758 (22.2) | 53038 |
| Male | Included with dated follow-up | 7899 (84.1) | 9390 |
| Female | Included with dated follow-up | 1491 (15.9) | 9390 |
| BMI category |  |  |  |
| Normal weight | Excluded without dated follow-up | 23367 (44.1) | 53038 |
| Overweight | Excluded without dated follow-up | 29671 (55.9) | 53038 |
| Normal weight | Included with dated follow-up | 3606 (38.4) | 9390 |
| Overweight | Included with dated follow-up | 5784 (61.6) | 9390 |
| Occupation |  |  |  |
| Customer service | Excluded without dated follow-up | 1135 (2.1) | 53038 |
| Health professionals | Excluded without dated follow-up | 2129 (4) | 53038 |
| Social services | Excluded without dated follow-up | 1236 (2.3) | 53038 |
| Physical work | Excluded without dated follow-up | 29393 (55.4) | 53038 |
| Office work | Excluded without dated follow-up | 19145 (36.1) | 53038 |
| Customer service | Included with dated follow-up | 66 (0.7) | 9390 |
| Health professionals | Included with dated follow-up | 142 (1.5) | 9390 |
| Social services | Included with dated follow-up | 565 (6) | 9390 |
| Physical work | Included with dated follow-up | 5173 (55.1) | 9390 |
| Office work | Included with dated follow-up | 3444 (36.7) | 9390 |

Table S3. Missing data by baseline variable.

| Characteristic | Missing (n) | Missing (%) | Completeness (%) |
| --- | --- | --- | --- |
| Age | 0 | 0 | 100.0 |
| Sex | 0 | 0 | 100.0 |
| Baseline BMI | 0 | 0 | 100.0 |
| Waist circumference | 1400 | 14.9 | 85.1 |
| Systolic blood pressure | 10 | 0.1 | 99.9 |
| Diastolic blood pressure | 14 | 0.1 | 99.9 |
| Glucose | 1478 | 15.7 | 84.3 |
| Total cholesterol | 4726 | 50.3 | 49.7 |
| Triglycerides | 4699 | 50.0 | 50.0 |
| Occupation type | 0 | 0 | 100.0 |
| Night work | 0 | 0 | 100.0 |
| Smoking | 0 | 0 | 100.0 |
| Alcohol consumption | 0 | 0 | 100.0 |
| Baseline diabetes | 1478 | 15.7 | 84.3 |
| Baseline prediabetes | 1626 | 17.3 | 82.7 |
| Dyslipidemia | 0 | 0 | 100.0 |

Table S4. Number at risk, events, and censoring by follow-up interval.

| Interval (months) | At risk at interval start | Interval events | Interval censoring | Cumulative events | Cumulative KM incidence (%) |
| --- | --- | --- | --- | --- | --- |
| 0-12 | 9390 | 35 | 19 | 35 | 0.4 |
| 12-24 | 9336 | 389 | 3072 | 424 | 5.5 |
| 24-36 | 5875 | 339 | 2991 | 763 | 13.1 |
| 36-48 | 2545 | 94 | 1040 | 857 | 17.2 |
| 48-60 | 1414 | 50 | 675 | 907 | 21.2 |
| 60-72 | 686 | 38 | 350 | 945 | 27.6 |
| 72-84 | 298 | 26 | 182 | 971 | 37.6 |
| 84-96 | 90 | 15 | 68 | 986 | 59.8 |

Table S5. Operational definition of variables.

| Characteristic | Definition | Role |
| --- | --- | --- |
| Baseline BMI | BMI valid from 10 to <80 kg/m²; primary cohort 18.5 to <30 kg/m² | Criterion/predictor |
| Incident obesity | First subsequent dated visit with BMI ≥30 kg/m² | Outcome |
| Time to event | Baseline date to detection; censored at the last valid visit with BMI <30 kg/m² | Time |
| Midpoint event date | Midpoint between the last visit with BMI <30 kg/m² and the first visit with BMI ≥30 kg/m² | Sensitivity |
| High baseline BP | SBP ≥140 or DBP ≥90 | Predictor |
| Laboratory variables | Baseline glucose, cholesterol, and triglycerides | Extended model |

Table S6. Specification of candidate prediction models.

| Model | Description | Continuous | Binary | Occupation | Note |
| --- | --- | --- | --- | --- | --- |
| M0_reference | Age + sex + baseline BMI | age10, baseline_bmi | male_sex | 0 |  |
| M1_minimal_clinical | Age + sex + baseline BMI + waist circumference | age10, baseline_bmi, waist_circumference10 | male_sex | 0 |  |
| M2_occupational_clinical | Occupational-clinical model with waist circumference | age10, baseline_bmi, waist_circumference10 | male_sex, night_work, smoking, alcohol, high_baseline_bp | 1 |  |
| M3_extended_laboratory | Extended laboratory model | age10, baseline_bmi, waist_circumference10, glucose10, cholesterol10, triglycerides10 | male_sex, night_work, smoking, alcohol, high_baseline_bp, diabetes, prediabetes | 1 |  |
| M4_without_waist_circumference | Occupational-clinical model without waist circumference | age10, baseline_bmi | male_sex, night_work, smoking, alcohol, high_baseline_bp | 1 |  |
| M5_ridge_penalized_Cox | Ridge-penalized Cox | M2 predictors; standardized | M2 indicators; standardized | 1 | λ=0.005 by 5-fold CV; apparent C-index=0.857; mean CV C-index=0.853 |

Table S7. Complete coefficients of candidate models.

| Model | Predictor | Model variable | Log-HR coefficient | HR |
| --- | --- | --- | --- | --- |
| M0_reference | Baseline age (per 10 years) | age10_c | -0,339 | 0,712 |
| M0_reference | Baseline BMI (per 1 kg/m²) | baseline_bmi_c | 0,730 | 2,075 |
| M0_reference | Male sex | male_sex | -0,194 | 0,824 |
| M1_minimal_clinical | Baseline age (per 10 years) | age10_c | -0,383 | 0,682 |
| M1_minimal_clinical | Baseline BMI (per 1 kg/m²) | baseline_bmi_c | 0,651 | 1,917 |
| M1_minimal_clinical | Baseline waist circumference (per 10 cm) | waist_circumference10_c | 0,344 | 1,411 |
| M1_minimal_clinical | Male sex | male_sex | -0,328 | 0,720 |
| M2_occupational_clinical | Baseline age (per 10 years) | age10_c | -0,365 | 0,694 |
| M2_occupational_clinical | Baseline BMI (per 1 kg/m²) | baseline_bmi_c | 0,647 | 1,909 |
| M2_occupational_clinical | Baseline waist circumference (per 10 cm) | waist_circumference10_c | 0,364 | 1,439 |
| M2_occupational_clinical | Male sex | male_sex | -0,421 | 0,657 |
| M2_occupational_clinical | Night work | night_work | 0,349 | 1,418 |
| M2_occupational_clinical | Baseline smoking | smoking | -0,038 | 0,963 |
| M2_occupational_clinical | Baseline alcohol use | alcohol | 0,041 | 1,042 |
| M2_occupational_clinical | High baseline blood pressure | high_baseline_bp | -0,244 | 0,783 |
| M2_occupational_clinical | Occupation: physical work vs office work | occupation_Physical_work | 0,242 | 1,274 |
| M2_occupational_clinical | Occupation: customer service vs office work | occupation_Customer_service | -0,128 | 0,880 |
| M2_occupational_clinical | Occupation: health professionals vs office work | occupation_Health_professionals | 0,235 | 1,265 |
| M2_occupational_clinical | Occupation: social services vs office work | occupation_Social_services | -0,516 | 0,597 |
| M3_extended_laboratory | Baseline age (per 10 years) | age10_c | -0,329 | 0,720 |
| M3_extended_laboratory | Baseline BMI (per 1 kg/m²) | baseline_bmi_c | 0,696 | 2,007 |
| M3_extended_laboratory | Baseline waist circumference (per 10 cm) | waist_circumference10_c | 0,266 | 1,304 |
| M3_extended_laboratory | Baseline glucose (per 10 mg/dL) | glucose10_c | -0,116 | 0,891 |
| M3_extended_laboratory | Baseline cholesterol (per 10 mg/dL) | cholesterol10_c | -0,002 | 0,998 |
| M3_extended_laboratory | Baseline triglycerides (per 10 mg/dL) | triglycerides10_c | -0,009 | 0,991 |
| M3_extended_laboratory | Male sex | male_sex | -0,311 | 0,733 |
| M3_extended_laboratory | Night work | night_work | 0,509 | 1,663 |
| M3_extended_laboratory | Baseline smoking | smoking | -0,049 | 0,952 |
| M3_extended_laboratory | Baseline alcohol use | alcohol | 0,099 | 1,104 |
| M3_extended_laboratory | High baseline blood pressure | high_baseline_bp | -0,359 | 0,699 |
| M3_extended_laboratory | Baseline prediabetes | prediabetes | 0,559 | 1,749 |
| M3_extended_laboratory | Occupation: physical work vs office work | occupation_Physical_work | 0,097 | 1,102 |
| M3_extended_laboratory | Occupation: customer service vs office work | occupation_Customer_service | 0,247 | 1,280 |
| M3_extended_laboratory | Occupation: health professionals vs office work | occupation_Health_professionals | -1,563 | 0,210 |
| M3_extended_laboratory | Occupation: social services vs office work | occupation_Social_services | -0,230 | 0,795 |
| M4_without_waist_circumference | Baseline age (per 10 years) | age10_c | -0,318 | 0,727 |
| M4_without_waist_circumference | Baseline BMI (per 1 kg/m²) | baseline_bmi_c | 0,731 | 2,076 |
| M4_without_waist_circumference | Male sex | male_sex | -0,271 | 0,763 |
| M4_without_waist_circumference | Night work | night_work | 0,361 | 1,434 |
| M4_without_waist_circumference | Baseline smoking | smoking | 0,004 | 1,004 |
| M4_without_waist_circumference | Baseline alcohol use | alcohol | -0,007 | 0,993 |
| M4_without_waist_circumference | High baseline blood pressure | high_baseline_bp | -0,478 | 0,620 |
| M4_without_waist_circumference | Occupation: physical work vs office work | occupation_Physical_work | 0,154 | 1,166 |
| M4_without_waist_circumference | Occupation: customer service vs office work | occupation_Customer_service | -0,671 | 0,511 |
| M4_without_waist_circumference | Occupation: health professionals vs office work | occupation_Health_professionals | 0,323 | 1,382 |
| M4_without_waist_circumference | Occupation: social services vs office work | occupation_Social_services | -0,339 | 0,712 |

Table S8. Summary of internal bootstrap validation.

| Model | B attempted | B successful | Apparent C-index | Mean optimism | Optimism-corrected C-index |
| --- | --- | --- | --- | --- | --- |
| M2_occupational_clinical | 504 | 500 | 0.857 | 0.00184 | 0.855 |

Table S8b. Implementation details of bootstrap internal validation.

| Item | Value | Comment |
| --- | --- | --- |
| Successful bootstrap replicates | 500 | Aggregate 500-replicate bootstrap output is summarized in Table S8. |
| Attempted bootstrap resamples | 504 | Four attempted resamples did not yield successful model fits and were skipped. |

Table S9. Calibration by deciles of 36-month predicted risk.

| Decile | n | Events | Mean predicted risk (%) | Observed KM 36-month incidence (%) | Predicted range (%) |
| --- | --- | --- | --- | --- | --- |
| (-0,001, 0,004] | 798 | 3 | 0.2 | 0 | 0-0.4 |
| (0,004, 0.01] | 798 | 7 | 0.7 | 1.6 | 0.4-1 |
| (0.01, 0,019] | 798 | 9 | 1.4 | 0.9 | 1-1.9 |
| (0,019, 0,034] | 798 | 10 | 2.6 | 1.1 | 1.9-3.4 |
| (0,034, 0,057] | 798 | 25 | 4.5 | 5.2 | 3.4-5.7 |
| (0,057, 0,095] | 797 | 34 | 7.4 | 6.2 | 5.7-9.5 |
| (0,095, 0,153] | 798 | 62 | 12.0 | 10.6 | 9.5-15.3 |
| (0,153, 0,252] | 798 | 99 | 19.8 | 18.2 | 15.3-25.2 |
| (0,252, 0,409] | 798 | 223 | 32.5 | 36.5 | 25.2-40.9 |
| (0,409, 0,972] | 798 | 361 | 57.2 | 59.3 | 40.9-97.2 |

Table S10. Net benefit by 36-month risk thresholds.

| Risk threshold | Final model | BMI reference model | Treat all | Treat none |
| --- | --- | --- | --- | --- |
| 0.0 | 0.26 | 0.26 | 0.26 | 0 |
| 0.0 | 0.26 | 0.26 | 0.26 | 0 |
| 0.0 | 0.25 | 0.25 | 0.25 | 0 |
| 0.0 | 0.25 | 0.24 | 0.24 | 0 |
| 0.1 | 0.24 | 0.24 | 0.23 | 0 |
| 0.1 | 0.23 | 0.23 | 0.23 | 0 |
| 0.1 | 0.23 | 0.23 | 0.22 | 0 |
| 0.1 | 0.23 | 0.22 | 0.21 | 0 |
| 0.1 | 0.22 | 0.22 | 0.20 | 0 |
| 0.1 | 0.22 | 0.22 | 0.19 | 0 |
| 0.1 | 0.22 | 0.21 | 0.18 | 0 |
| 0.1 | 0.21 | 0.21 | 0.17 | 0 |
| 0.1 | 0.21 | 0.20 | 0.16 | 0 |
| 0.1 | 0.20 | 0.20 | 0.15 | 0 |
| 0.1 | 0.20 | 0.20 | 0.14 | 0 |
| 0.2 | 0.20 | 0.19 | 0.13 | 0 |
| 0.2 | 0.20 | 0.19 | 0.12 | 0 |
| 0.2 | 0.19 | 0.19 | 0.11 | 0 |
| 0.2 | 0.19 | 0.18 | 0.10 | 0 |
| 0.2 | 0.18 | 0.18 | 0.09 | 0 |
| 0.2 | 0.18 | 0.17 | 0.08 | 0 |
| 0.2 | 0.18 | 0.17 | 0.07 | 0 |
| 0.2 | 0.17 | 0.16 | 0.05 | 0 |
| 0.2 | 0.17 | 0.16 | 0.04 | 0 |
| 0.2 | 0.16 | 0.15 | 0.03 | 0 |
| 0.3 | 0.16 | 0.15 | 0.02 | 0 |
| 0.3 | 0.15 | 0.14 | 0.00 | 0 |
| 0.3 | 0.15 | 0.14 | -0.01 | 0 |
| 0.3 | 0.15 | 0.14 | -0.03 | 0 |
| 0.3 | 0.14 | 0.13 | -0.04 | 0 |

Table S11. Conversion of score points to estimated absolute risk.

| Score points | n | Mean predicted 36-month risk (%) | Events | Observed KM 36-month incidence (%) |
| --- | --- | --- | --- | --- |
| 0 | 1 | 0.0 | 0 |  |
| 1 | 2 | 0.0 | 0 |  |
| 2 | 6 | 0.0 | 0 |  |
| 3 | 9 | 0.0 | 0 |  |
| 4 | 28 | 0.1 | 0 | 0 |
| 5 | 33 | 0.1 | 0 | 0 |
| 6 | 47 | 0.1 | 0 | 0 |
| 7 | 53 | 0.1 | 1 | 0 |
| 8 | 77 | 0.1 | 0 | 0 |
| 9 | 85 | 0.1 | 0 | 0 |
| 10 | 90 | 0.2 | 0 | 0 |
| 11 | 104 | 0.2 | 0 | 0 |
| 12 | 132 | 0.3 | 0 | 0 |
| 13 | 132 | 0.4 | 3 | 2.9 |
| 14 | 186 | 0.5 | 1 | 1.9 |
| 15 | 187 | 0.6 | 3 | 2.1 |
| 16 | 224 | 0.7 | 2 | 1.0 |
| 17 | 206 | 0.9 | 1 | 0 |
| 18 | 241 | 1.1 | 0 | 0 |
| 19 | 287 | 1.4 | 3 | 1.4 |
| 20 | 284 | 1.7 | 6 | 1.2 |
| 21 | 265 | 2.2 | 3 | 1.2 |
| 22 | 334 | 2.7 | 7 | 3.1 |
| 23 | 309 | 3.4 | 8 | 2.6 |
| 24 | 325 | 4.2 | 5 | 1.6 |
| 25 | 332 | 5.2 | 17 | 9.6 |
| 26 | 363 | 6.5 | 13 | 4.7 |
| 27 | 337 | 8.0 | 13 | 7.3 |
| 28 | 377 | 10.1 | 19 | 6.4 |
| 29 | 358 | 12.2 | 27 | 9.2 |
| 30 | 313 | 15.3 | 26 | 12.2 |
| 31 | 314 | 18.6 | 34 | 16.1 |
| 32 | 318 | 22.8 | 50 | 24.1 |
| 33 | 290 | 27.1 | 62 | 28.6 |
| 34 | 325 | 33.1 | 103 | 39.5 |
| 35 | 280 | 39.2 | 85 | 39.4 |
| 36 | 225 | 46.6 | 90 | 55.8 |
| 37 | 221 | 55.6 | 101 | 56.1 |
| 38 | 165 | 64.2 | 88 | 67.6 |
| 39 | 71 | 73.7 | 35 | 61.0 |
| 40 | 31 | 82.6 | 19 | 75.2 |
| 41 | 11 | 88.6 | 8 | 100 |
| 43 | 1 | 97.2 | 0 |  |

Table S12. Tabular score calculator.

| Predictor | Model variable | Points per unit/category |
| --- | --- | --- |
| Baseline age (per 10 years) | age10_c | -1 |
| Baseline BMI (per 1 kg/m²) | baseline_bmi_c | 3 |
| Baseline waist circumference (per 10 cm) | waist_circumference10_c | 1 |
| Male sex | male_sex | -2 |
| Night work | night_work | 1 |
| Baseline smoking | smoking | 0 |
| Baseline alcohol use | alcohol | 0 |
| High baseline blood pressure | high_baseline_bp | -1 |
| Occupation: physical work vs office work | occupation_Physical_work | 1 |
| Occupation: customer service vs office work | occupation_Customer_service | -1 |
| Occupation: health professionals vs office work | occupation_Health_professionals | 1 |
| Occupation: social services vs office work | occupation_Social_services | -2 |
| Shift | score | Subtract 56 from the raw total |

Table S13. Sensitivity analysis using the midpoint as the event date.

| Model | Description | n | Events | Effective predictors | Events per predictor | Apparent C-index | 36-month IPCW Brier | 0-36-month IPCW IBS | n with known 36-month status | 36-month AUC among those with known status | 36-month Brier among those with known status |
| --- | --- | --- | --- | --- | --- | --- | --- | --- | --- | --- | --- |
| Midpoint_event_date_sensitivity | Occupational-clinical model with waist circumference | 7979 | 833 | 12 | 69.4 | 0.86 | 0,080 | 0.05 | 2522 | 0,884 | 0,157 |

Table S14. Fixed-horizon models and performance at 36 and 60 months.

| Horizon (months) | n with known status | Events | AUC of Cox-derived risk | Brier | Mean predicted risk (%) |
| --- | --- | --- | --- | --- | --- |
| 36 | 2522 | 685 | 0.9 | 0,135 | 18.7 |
| 60 | 1065 | 803 | 0.9 | 0,198 | 48.7 |

Table S15. Analysis by baseline BMI subcohorts.

| Subcohort | n | Events | Error | Model | Description | Effective predictors | Events per predictor | Apparent C-index | 36-month IPCW Brier | 0-36-month IPCW IBS | n with known 36-month status | 36-month AUC among those with known status | 36-month Brier among those with known status |
| --- | --- | --- | --- | --- | --- | --- | --- | --- | --- | --- | --- | --- | --- |
| Baseline normal weight | 3606 | Events | Singular matrix |  |  |  |  |  |  |  |  |  |  |
| Baseline overweight | 4902 | Events |  | Baseline_overweight_subcohort | Occupational-clinical model with waist circumference | 12 | 66.2 | 0.80 | 0,135 | 0.06 | 1731 | 0,823 | 0,184 |

Table S16. Model without waist circumference and descriptive waist-imputation analysis.

| Model | Description | n | Events | Effective predictors | Events per predictor | Apparent C-index | 36-month IPCW Brier | 0-36-month IPCW IBS | n with known 36-month status | 36-month AUC among those with known status | 36-month Brier among those with known status | Sensitivity |
| --- | --- | --- | --- | --- | --- | --- | --- | --- | --- | --- | --- | --- |
| M4_without_waist_circumference | Occupational-clinical model without waist circumference | 9376 | 986 | 11 | 89.6 | 0.85 | 0,086 | 0.04 | 3303 | 0,871 | 0,126 | Model without waist circumference |
| M2_waist_circumference_imputed | Occupational-clinical model with waist circumference | 9376 | 986 | 12 | 82.2 | 0.86 | 0,085 | 0.04 | 3303 | 0,876 | 0,124 | Descriptive median imputation by sex x BMI category; not used for final inference. |

Table S17. Extended model with laboratory variables.

| Model | Description | n | Events | Effective predictors | Events per predictor | Apparent C-index | 36-month IPCW Brier | 0-36-month IPCW IBS | n with known 36-month status | 36-month AUC among those with known status | 36-month Brier among those with known status |
| --- | --- | --- | --- | --- | --- | --- | --- | --- | --- | --- | --- |
| M3_extended_laboratory | Extended laboratory model | 3219 | 311 | 16 | 19.4 | 0.86 | 0,074 | 0.03 | 1159 | 0,893 | 0,111 |

Table S18. Exclusion of early events and strict outcome definition.

| Model | Description | n | Events | Effective predictors | Events per predictor | Apparent C-index | 36-month IPCW Brier | 0-36-month IPCW IBS | n with known 36-month status | 36-month AUC among those with known status | 36-month Brier among those with known status | n excluded | Confirmed events |
| --- | --- | --- | --- | --- | --- | --- | --- | --- | --- | --- | --- | --- | --- |
| Exclude_events_before_6m | Occupational-clinical model with waist circumference | 7978 | 832 | 12 | 69.3 | 0.86 | 0,091 | 0.04 | 2521 | 0,878 | 0,135 | 3 |  |
| Exclude_events_before_12m | Occupational-clinical model with waist circumference | 7950 | 804 | 12 | 67 | 0.86 | 0,090 | 0.04 | 2493 | 0,878 | 0,134 | 35 |  |
| Subsequently_confirmed_event | Occupational-clinical model with waist circumference | 7979 | 109 | 12 | 9.08 |  |  |  | 1939 |  |  |  | 133 |

Table S19. Alternative rules for anthropometric outliers.

| Valid BMI rule | n | Events | Crude risk (%) |
| --- | --- | --- | --- |
| 10 to <80 | 9390 | 987 | 10.5 |
| 12 to <70 | 9390 | 987 | 10.5 |
| 15 to <60 | 9390 | 987 | 10.5 |

Table S20. Distribution of events by baseline year.

| Baseline year | n | Events | Crude risk (%) | Median follow-up (months) |
| --- | --- | --- | --- | --- |
| 2013 | 354 | 42 | 11.9 | 64.6 |
| 2014 | 1561 | 158 | 10.1 | 43.0 |
| 2015 | 1614 | 140 | 8.7 | 33.2 |
| 2016 | 997 | 91 | 9.1 | 27.8 |
| 2017 | 1487 | 165 | 11.1 | 25.0 |
| 2018 | 1315 | 160 | 12.2 | 25.2 |
| 2019 | 1744 | 194 | 11.1 | 23.2 |
| 2020 | 311 | 37 | 11.9 | 15.3 |
| 2021 | 7 | 0 | 0.0 | 14.0 |

Table S21. Temporal validation.

| Temporal cutoff | Development n | Development events | Validation n | Validation events | Validation C-index | 36-month validation Brier |
| --- | --- | --- | --- | --- | --- | --- |
| 2017-08-26 | 3993 | 381 | 3986 | 452 | 0.84 | 0,173 |

Table S22. Selection by availability of dated follow-up.

| Baseline eligible with date | With dated follow-up | Proportion with follow-up (%) | Note |
| --- | --- | --- | --- |
| 62428 | 9390 | 15.0 | Descriptive diagnosis of selection by reassessment |

Table S23. Frequency by occupational grouping.

| Occupation | n |
| --- | --- |
| Physical work | 5173 |
| Office work | 3444 |
| Social services | 565 |
| Health professionals | 142 |
| Customer service | 66 |

Table S24. Approximate diagnosis of the proportional hazards assumption.

| Predictor | Model variable | Spearman residual-log(time) |
| --- | --- | --- |
| Baseline age (per 10 years) | age10_c | 0,045 |
| Baseline BMI (per 1 kg/m²) | baseline_bmi_c | 0,023 |
| Baseline waist circumference (per 10 cm) | waist_circumference10_c | 0,078 |
| Male sex | male_sex | 0,037 |
| Night work | night_work | 0,025 |
| Baseline smoking | smoking | 0,055 |
| Baseline alcohol use | alcohol | -0,094 |
| High baseline blood pressure | high_baseline_bp | 0,071 |
| Occupation: physical work vs office work | occupation_Physical_work | -0,111 |
| Occupation: customer service vs office work | occupation_Customer_service | 0,017 |
| Occupation: health professionals vs office work | occupation_Health_professionals | -0,022 |
| Occupation: social services vs office work | occupation_Social_services | 0,074 |

Table S25. Predictive performance by subgroup.

| Subgroup | n | Events | C-index | 36-month Brier | Predicted 36-month risk (%) | 36-month KM incidence (%) |
| --- | --- | --- | --- | --- | --- | --- |
| Sex |  |  |  |  |  |  |
| Male | 6692 | 707 | 0.85 | 0,093 | 14.1 | 14.7 |
| Female | 1287 | 126 | 0.88 | 0,079 | 12.4 | 14.2 |
| Baseline BMI category |  |  |  |  |  |  |
| Normal weight | 3077 | 39 | 0.70 | 0,014 | 1.3 | 1.5 |
| Overweight | 4902 | 794 | 0.80 | 0,135 | 21.7 | 22.3 |
| Age |  |  |  |  |  |  |
| 30-39 | 2440 | 281 | 0.86 | 0,100 | 16.3 | 16.3 |
| 40-49 | 1636 | 168 | 0.84 | 0,095 | 14.7 | 14.8 |
| <30 | 2701 | 270 | 0.88 | 0,080 | 12.4 | 14.0 |
| ≥50 | 1202 | 114 | 0.82 | 0,091 | 10.9 | 12.4 |
| Occupation |  |  |  |  |  |  |
| Health professionals | 126 | 13 | 0.68 | 0,114 | 14.9 | 17.5 |
| Social services | 553 | 43 | 0.88 | 0,076 | 8.6 | 10.7 |
| Physical work | 4371 | 485 | 0.85 | 0,103 | 15.6 | 17.0 |
| Office work | 2879 | 289 | 0.87 | 0,079 | 12.2 | 12.4 |
| Night work |  |  |  |  |  |  |
| No | 7517 | 741 | 0.86 | 0,088 | 13.3 | 14.1 |
| Yes | 462 | 92 | 0.80 | 0,134 | 22.5 | 23.1 |

Table S26. Exploratory dynamic landmark model.

| n landmark | Future events | Median time to landmark (months) | Median BMI delta |
| --- | --- | --- | --- |
| 1456 | 111 | 12.4 | 0 |

**SUPPLEMENTARY FIGURES**

Figure S1. Flow diagram of the analytical cohort.


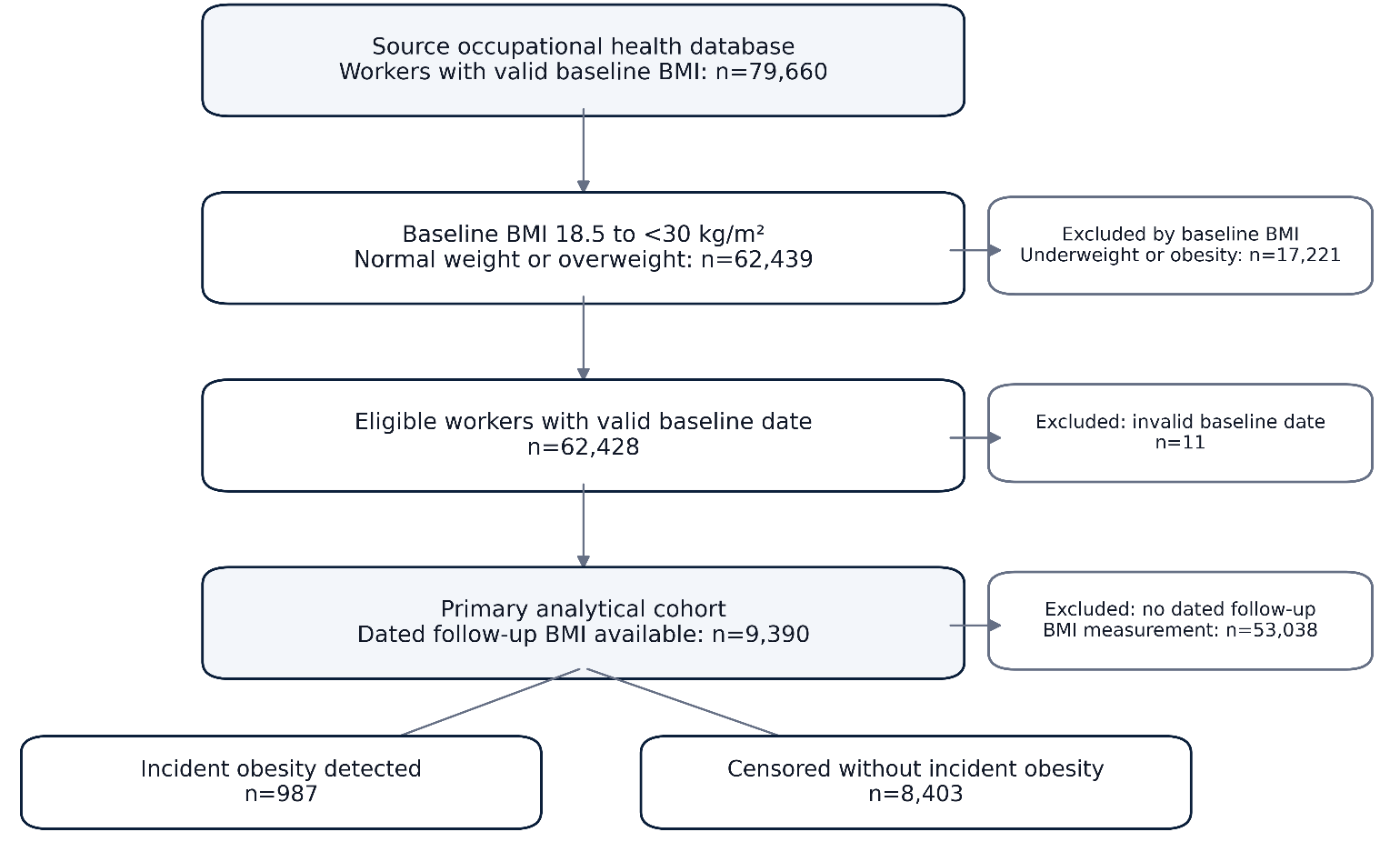


Figure S2. Missing-data pattern.


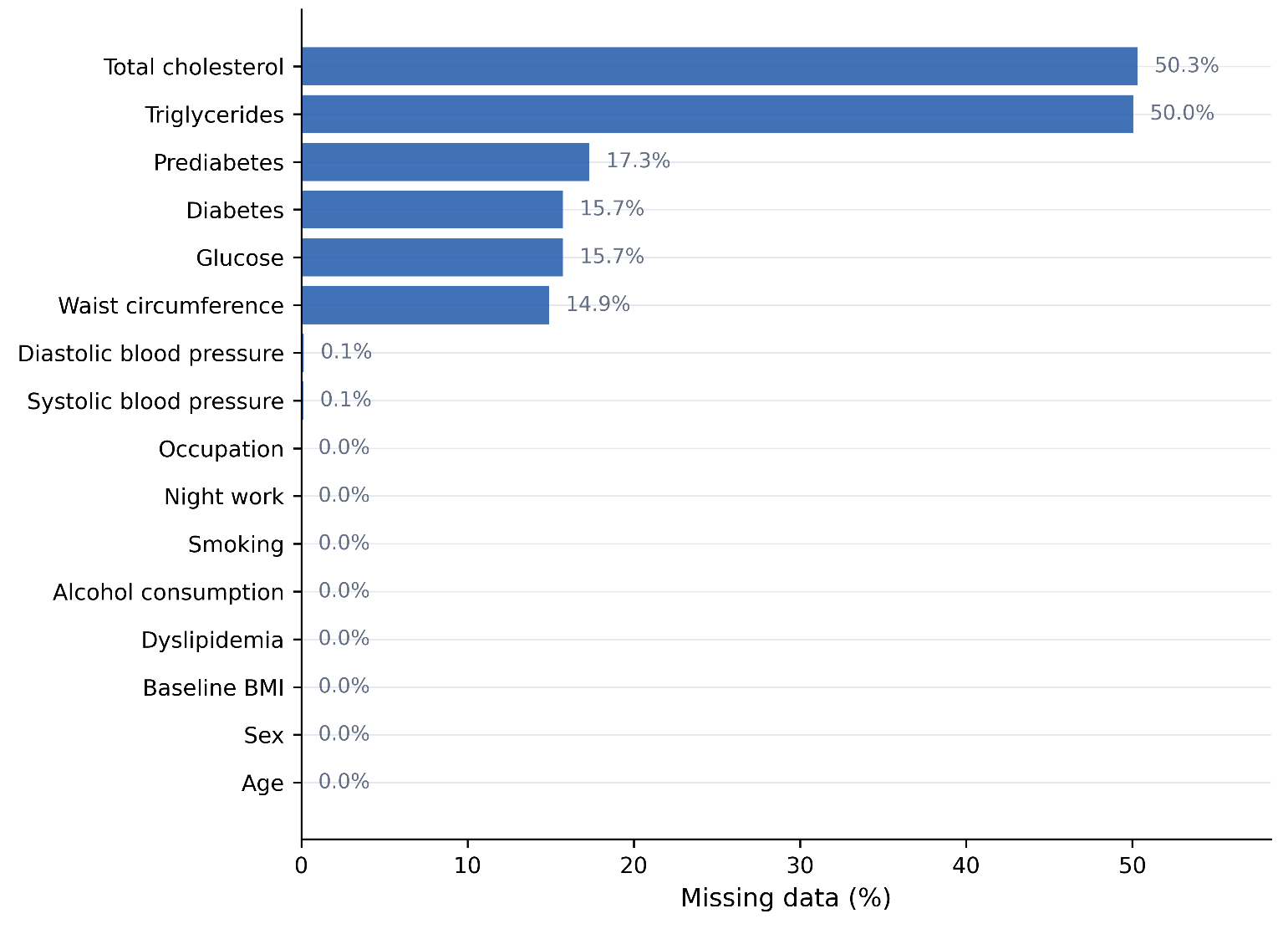


Figure S3. Distribution of the interval between occupational visits.


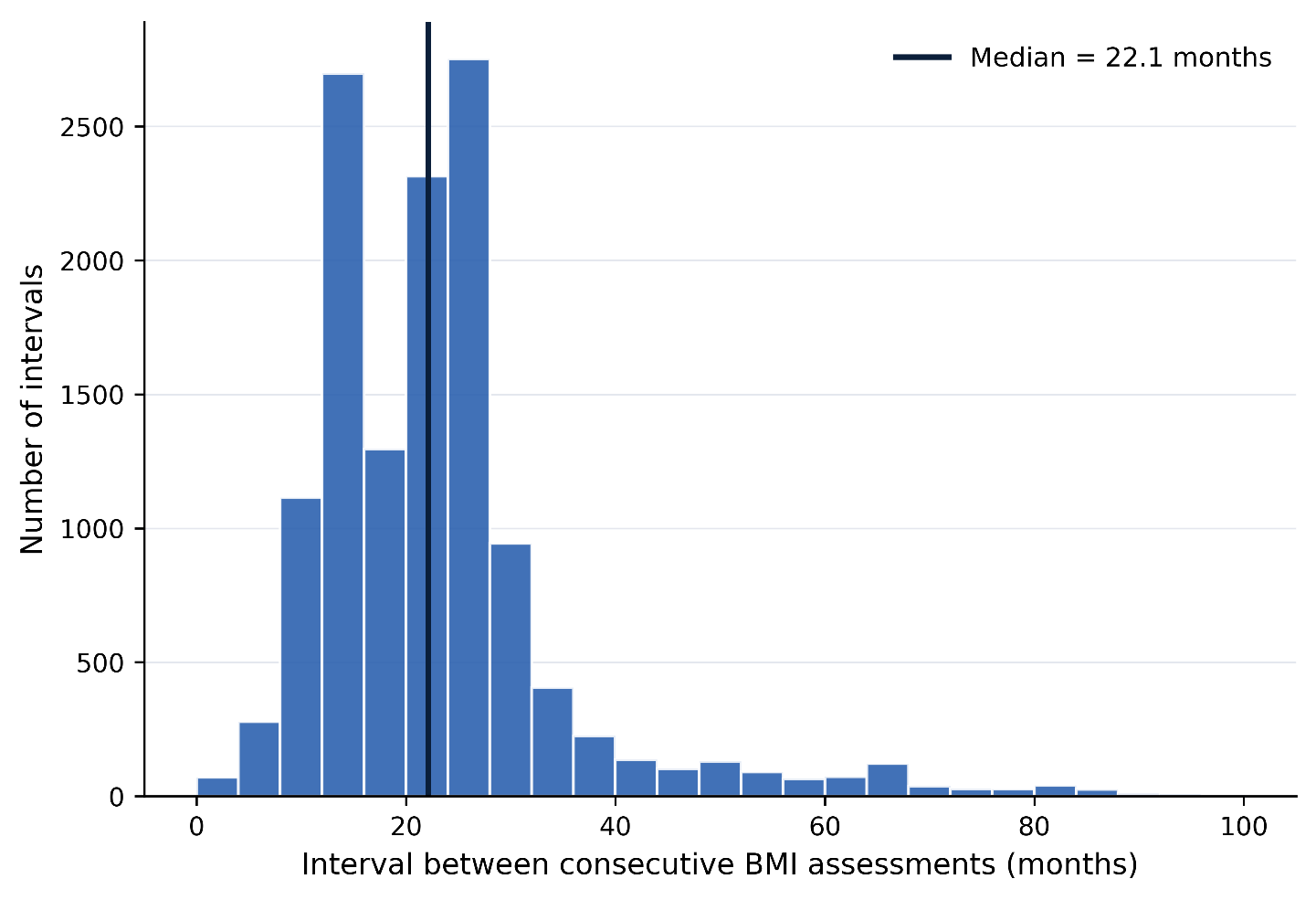


Figure S4. Distribution of total follow-up time.


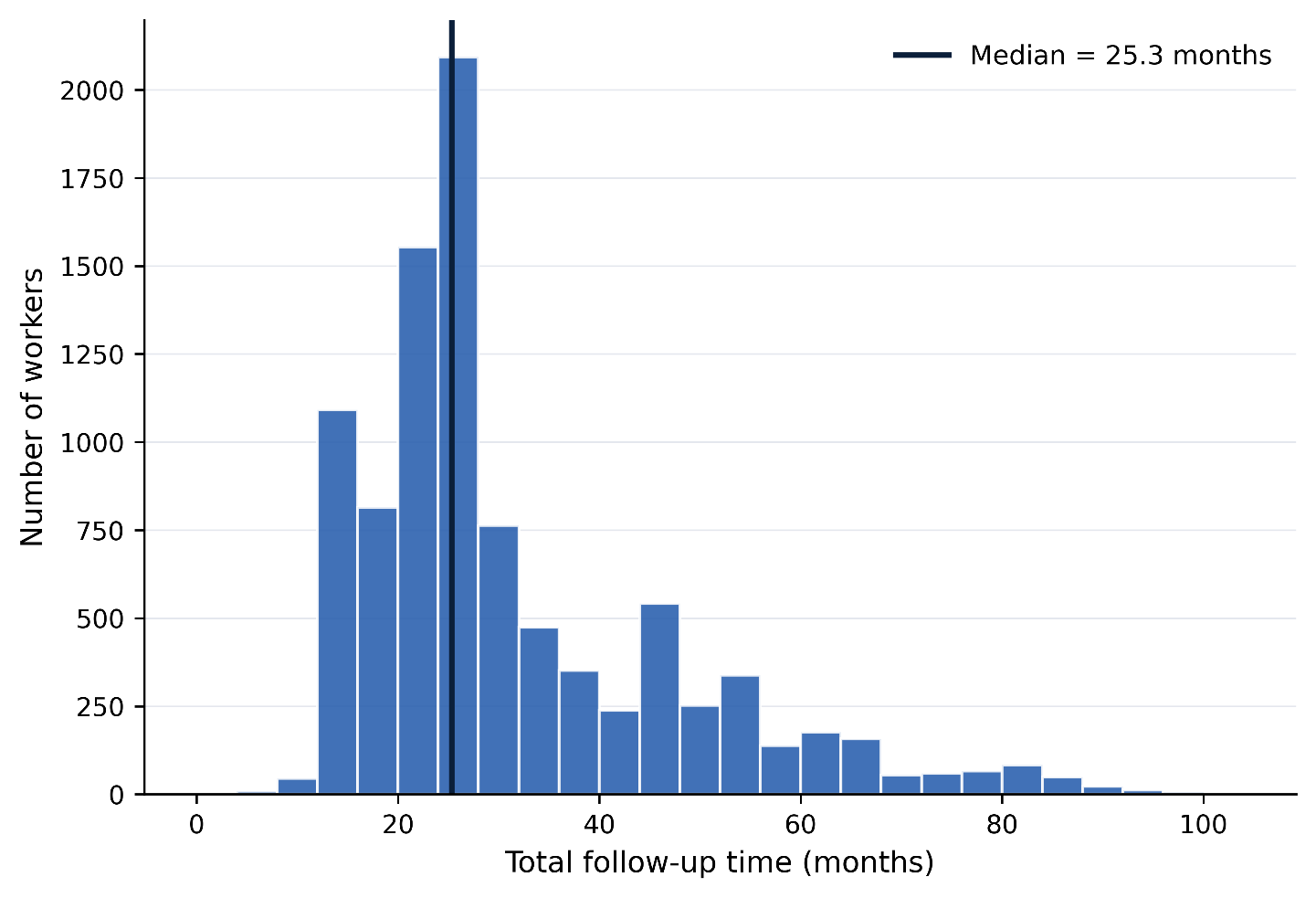


Figure S5. Coefficients of the final model.


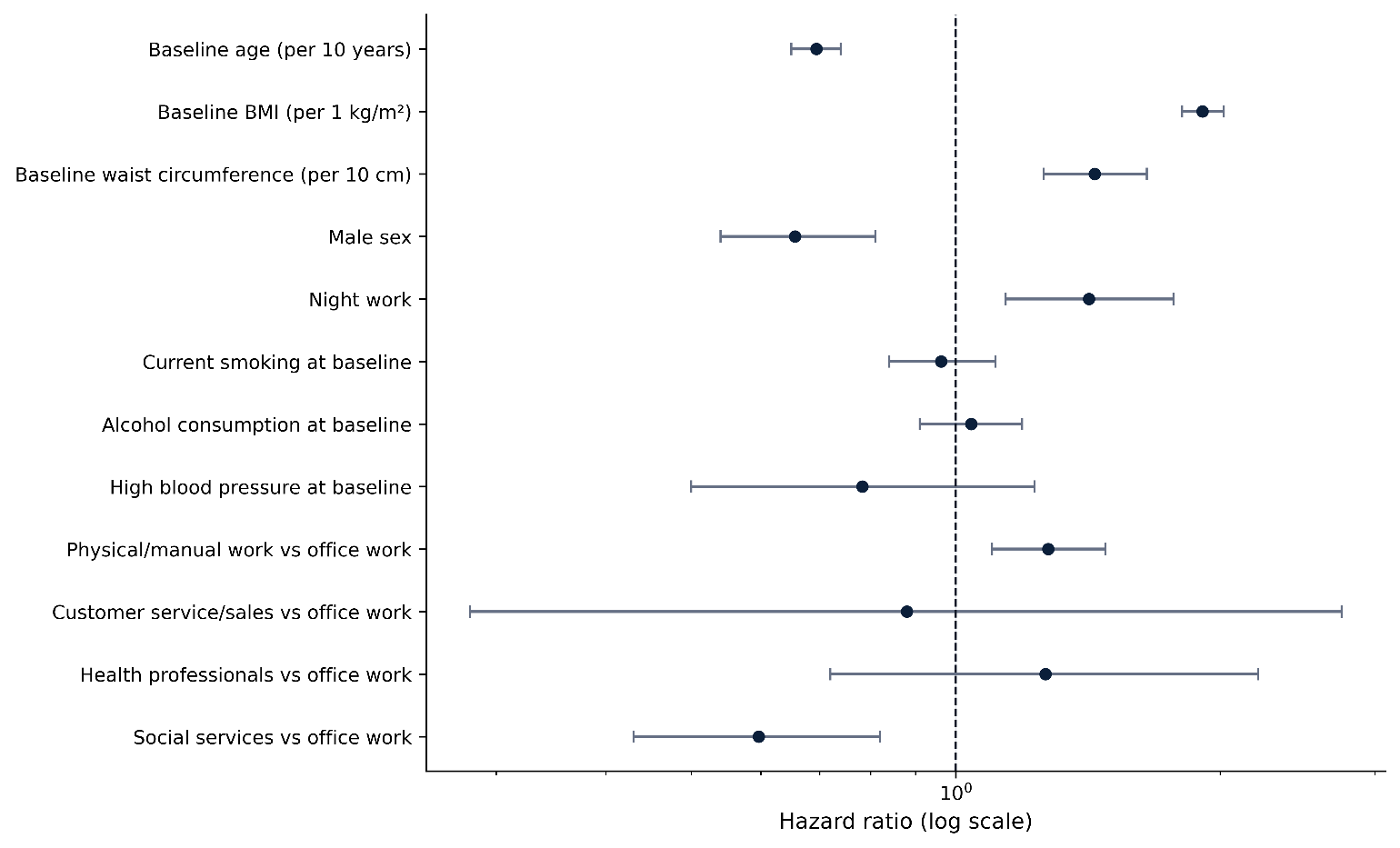


Figure S6. Summary of bootstrap internal validation.


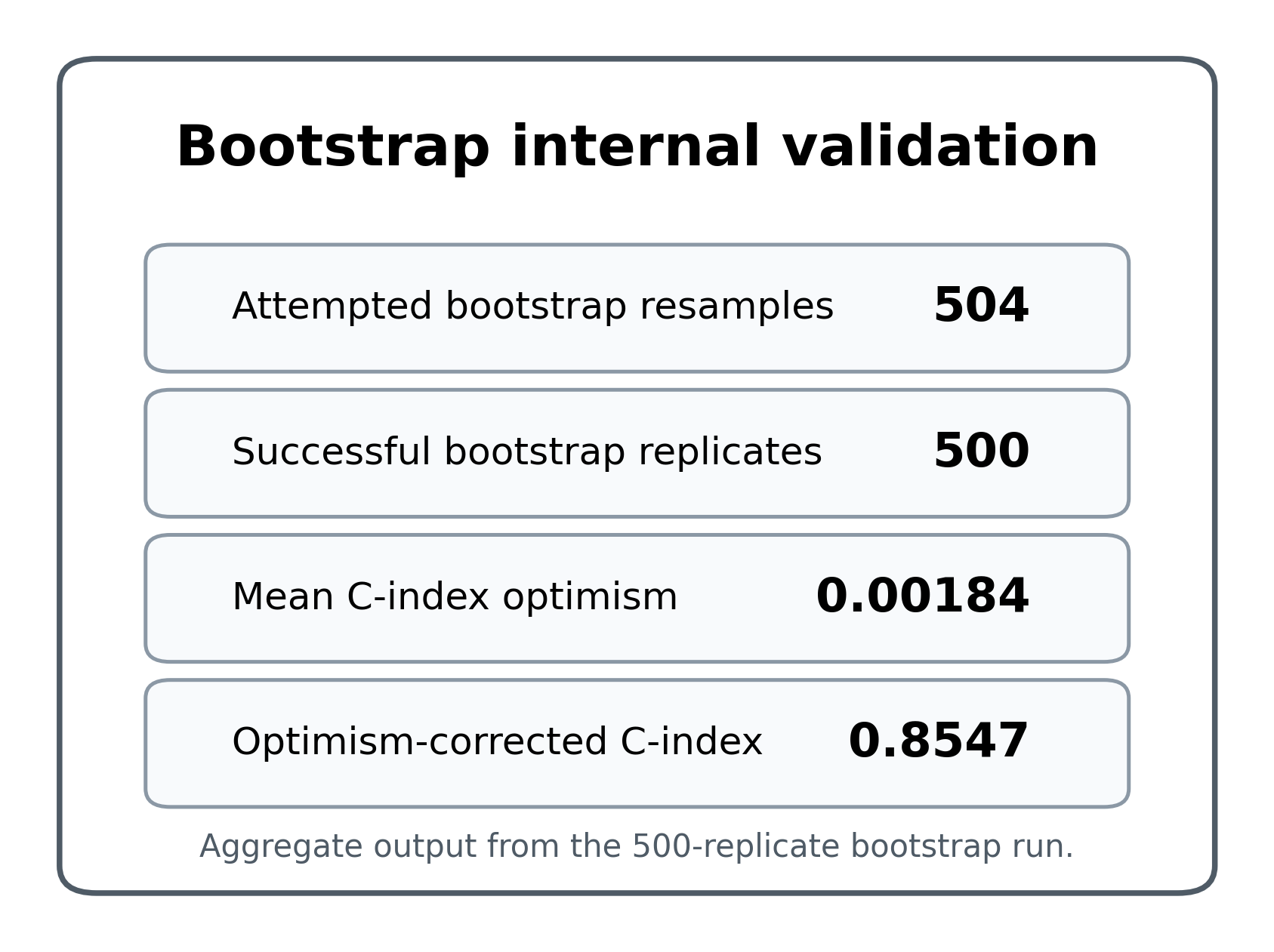


Figure S7. Calibration of the final model by baseline BMI category.


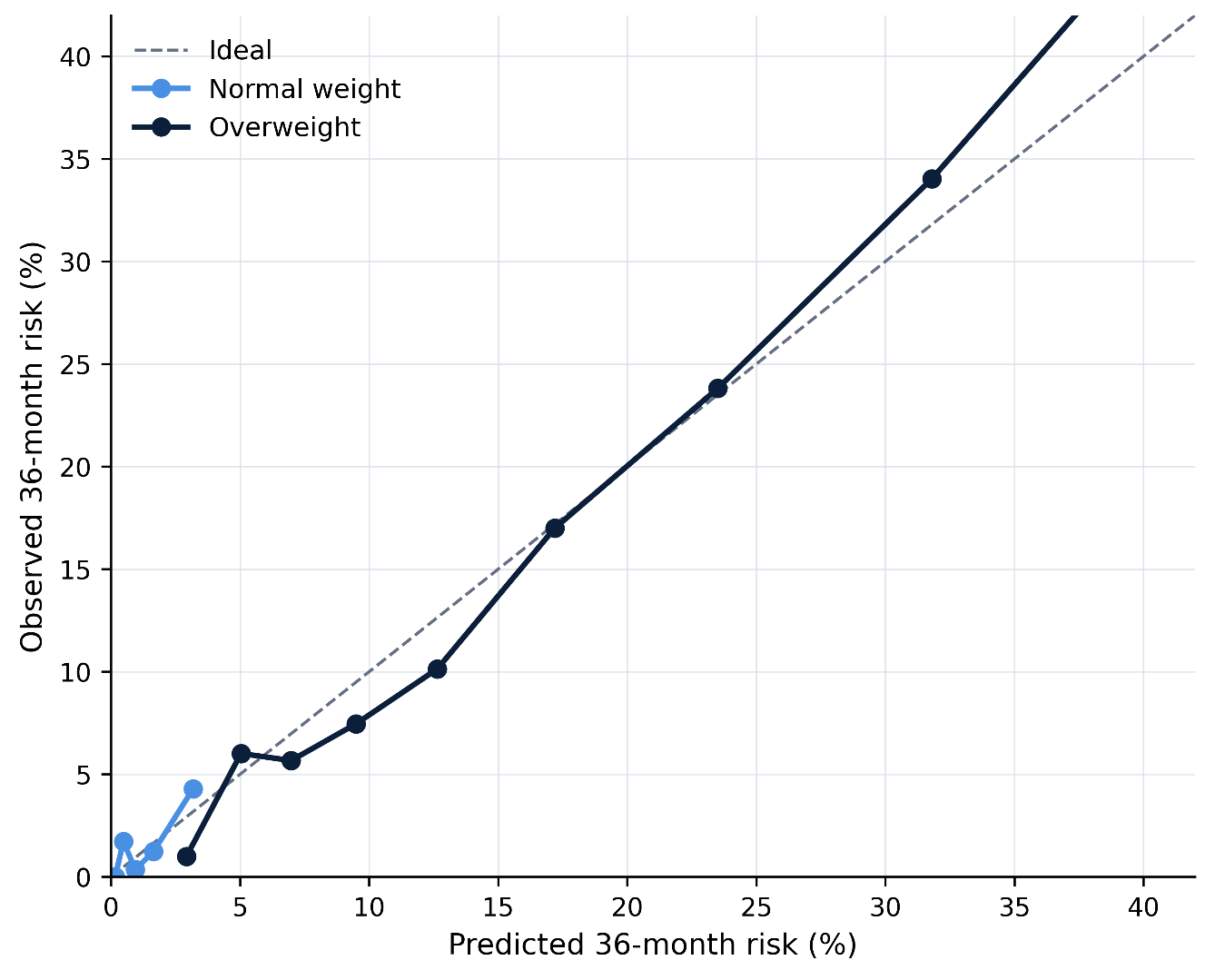


Figure S8. Exploratory calibration at 60 months.


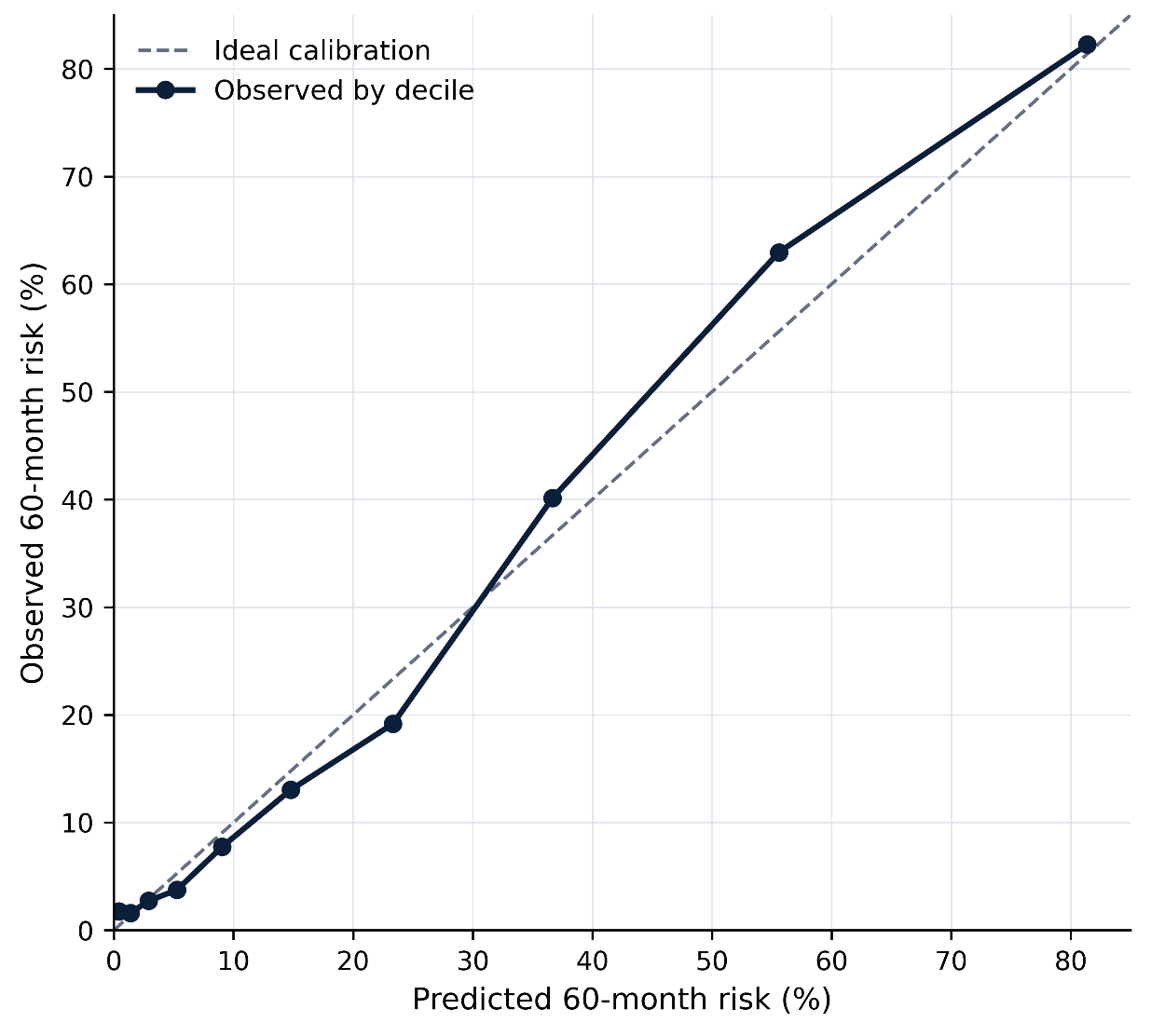


Figure S9. Net-benefit difference versus the reference model.


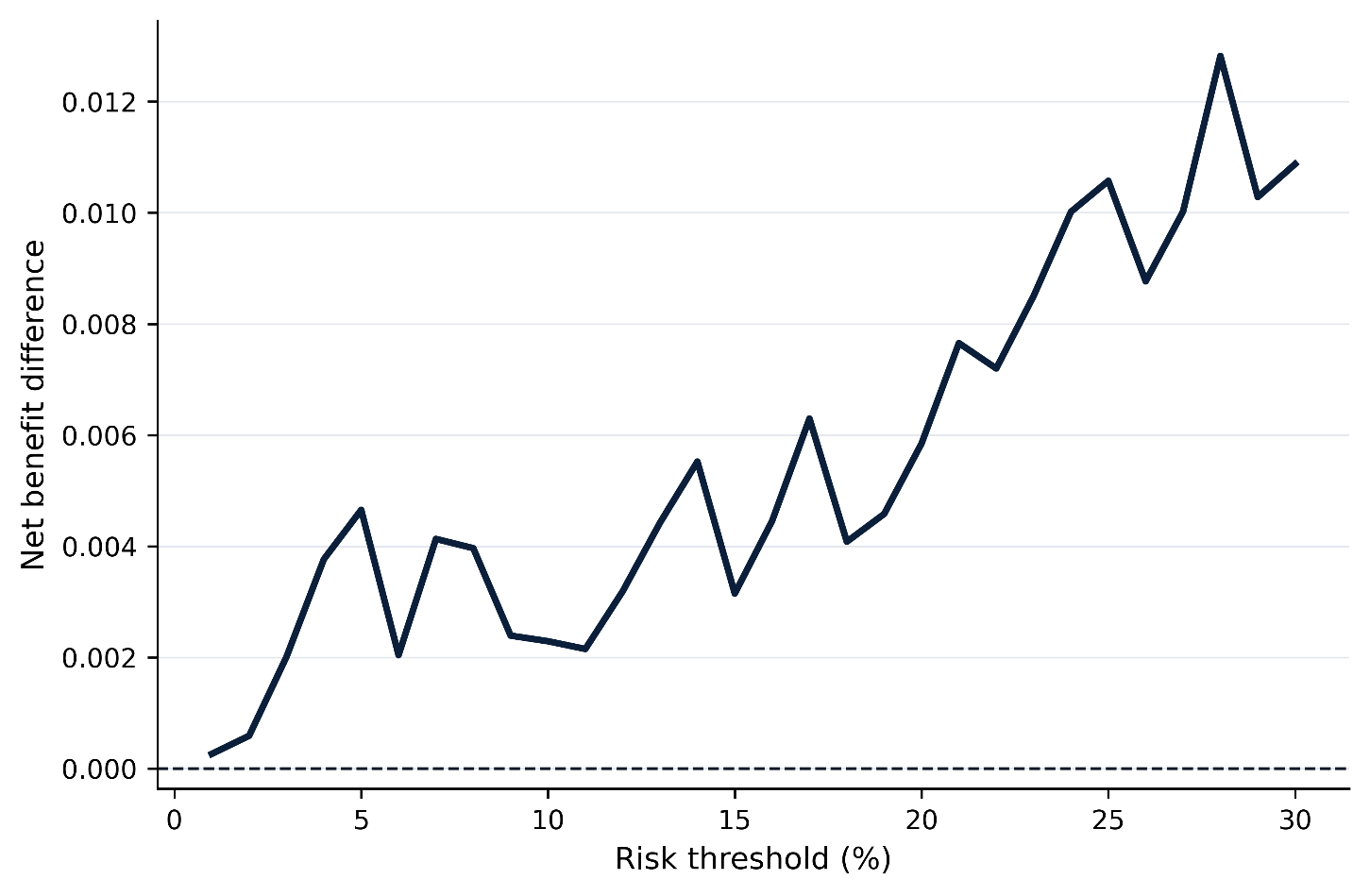


Figure S10. Distribution of the total score.


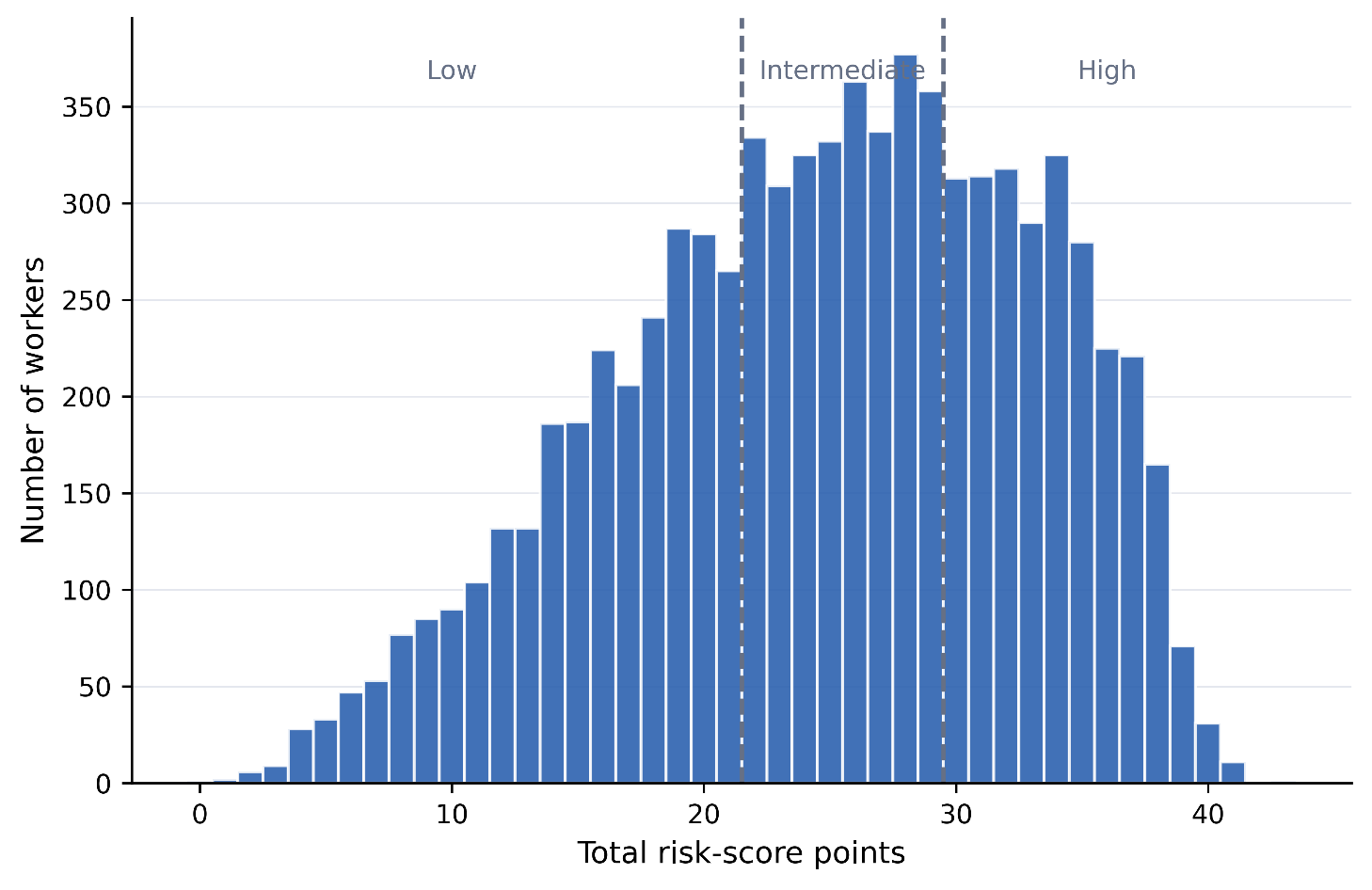


Figure S11. Observed and predicted risk by score deciles.


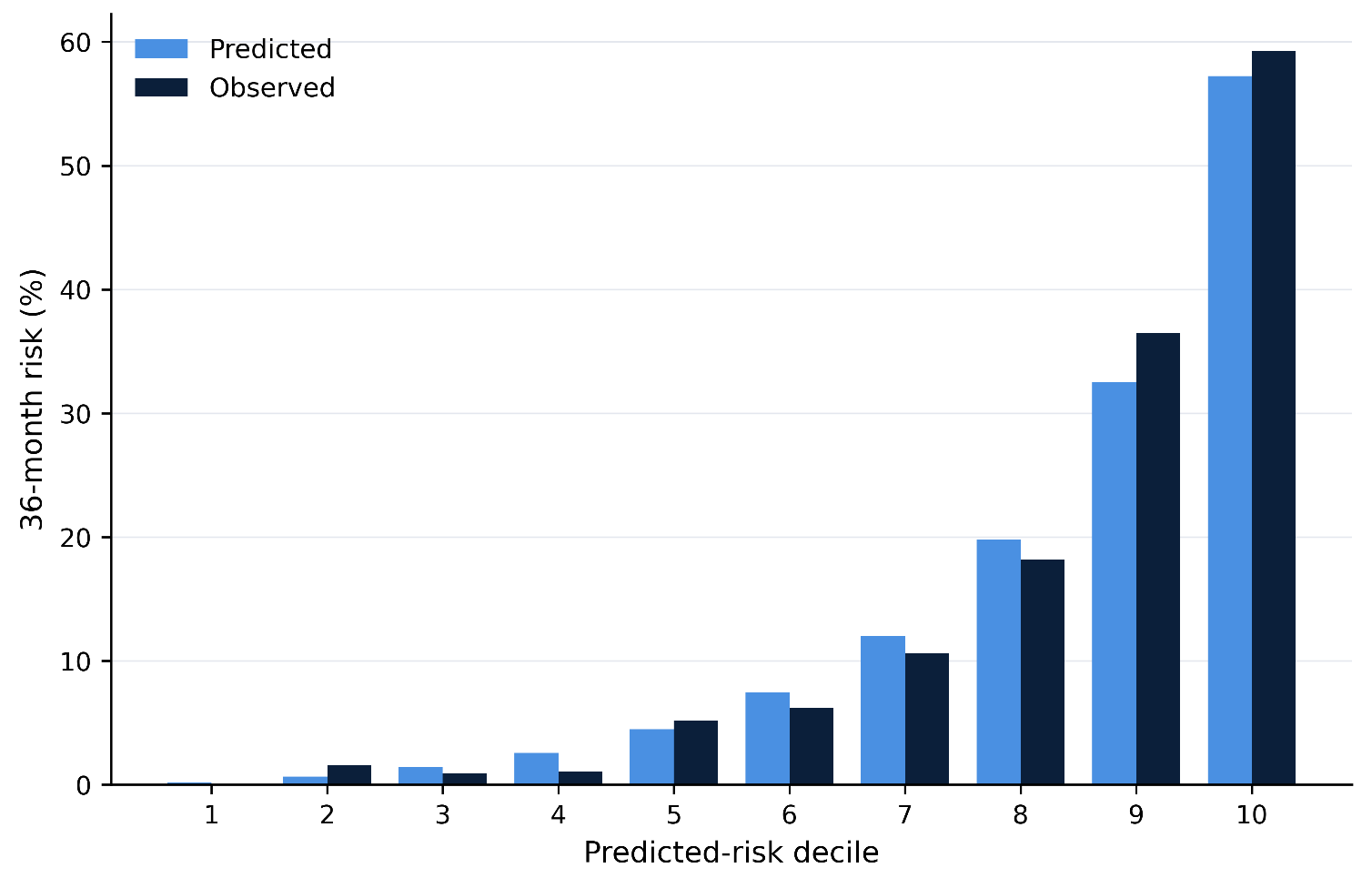


Figure S12. Sensitivity analysis using the midpoint as the event date.


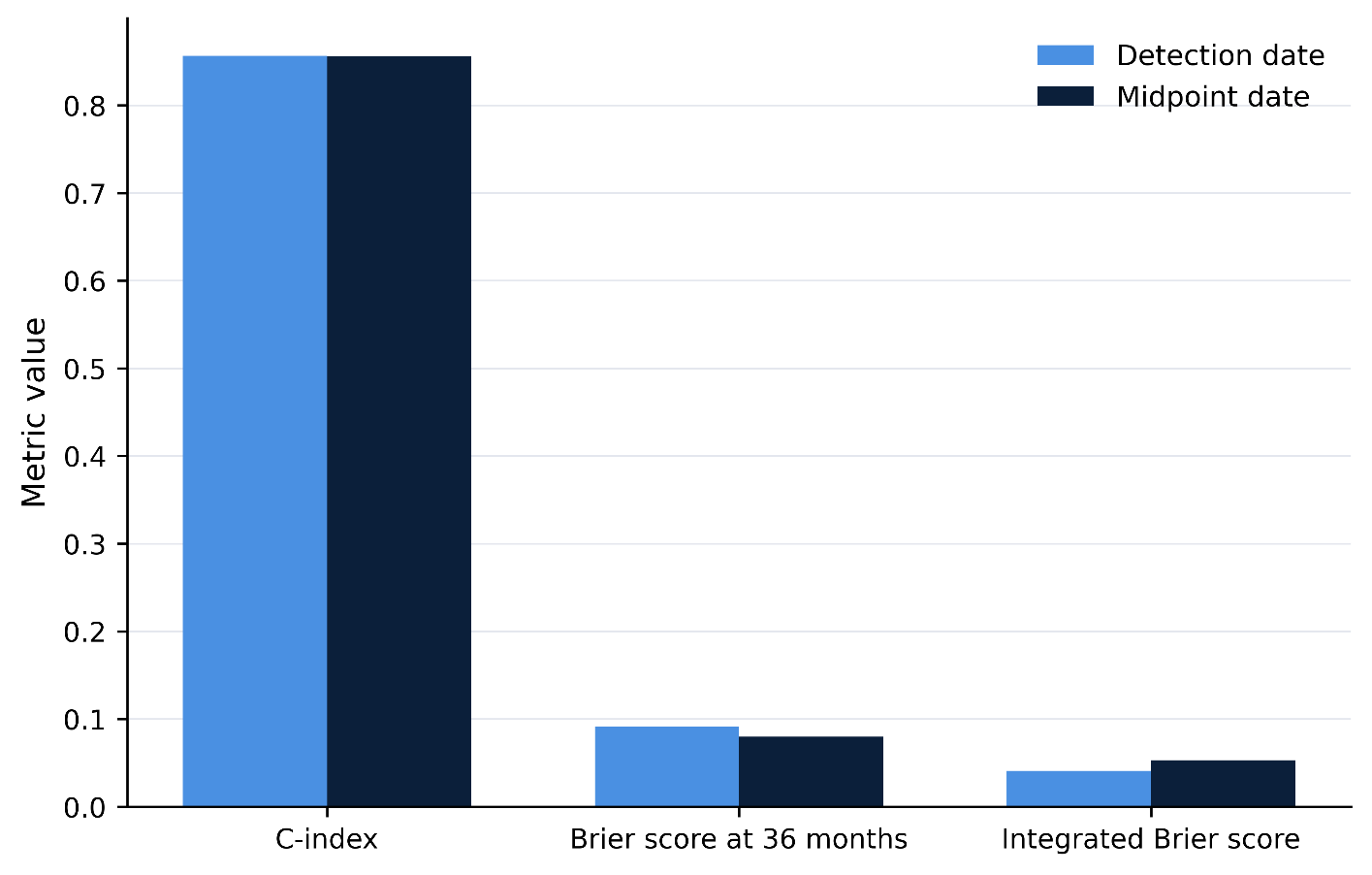


Figure S13. Calibration in the baseline overweight subcohort.


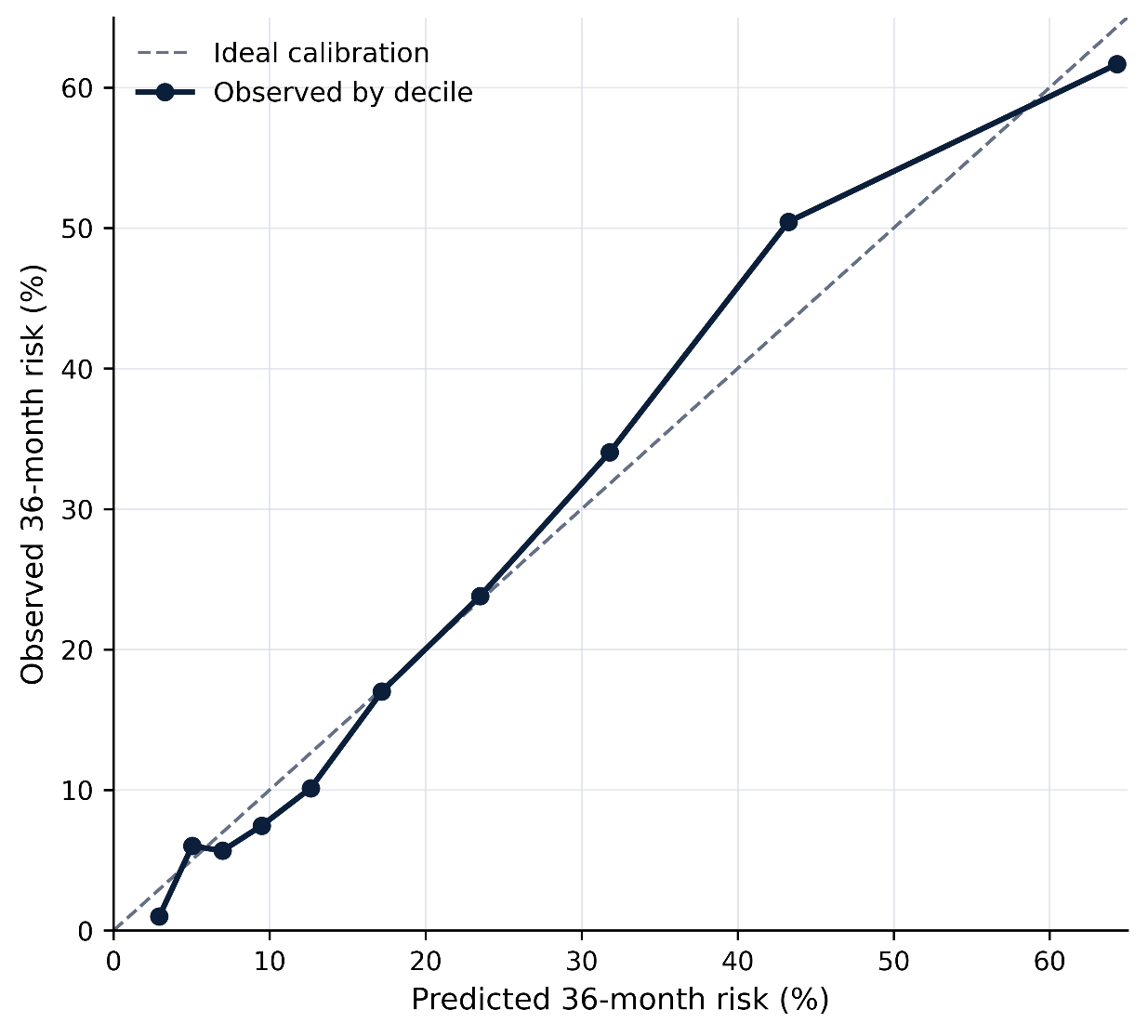


Figure S14. Calibration of the model without waist circumference.


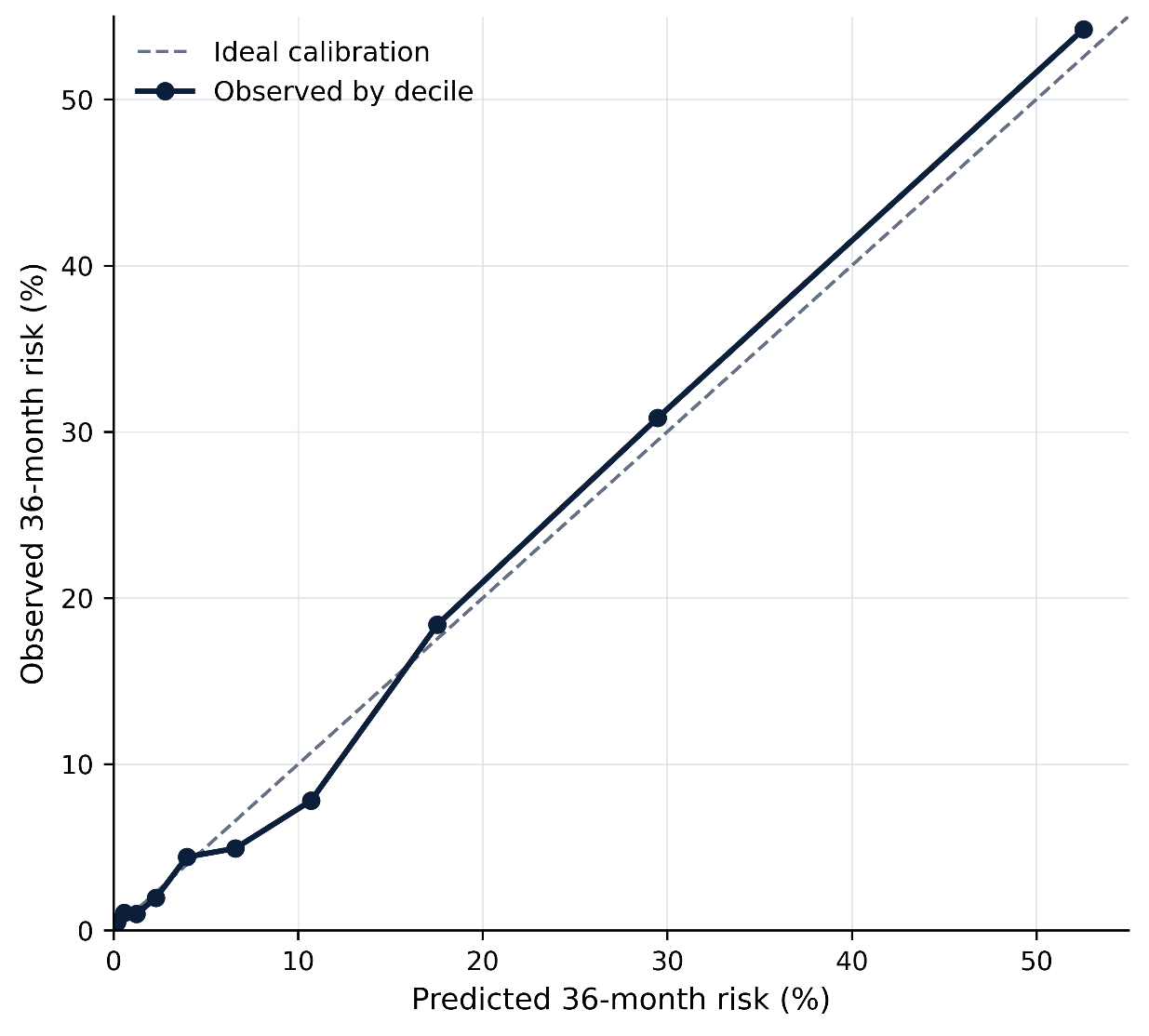


Figure S15. Calibration in temporal validation.


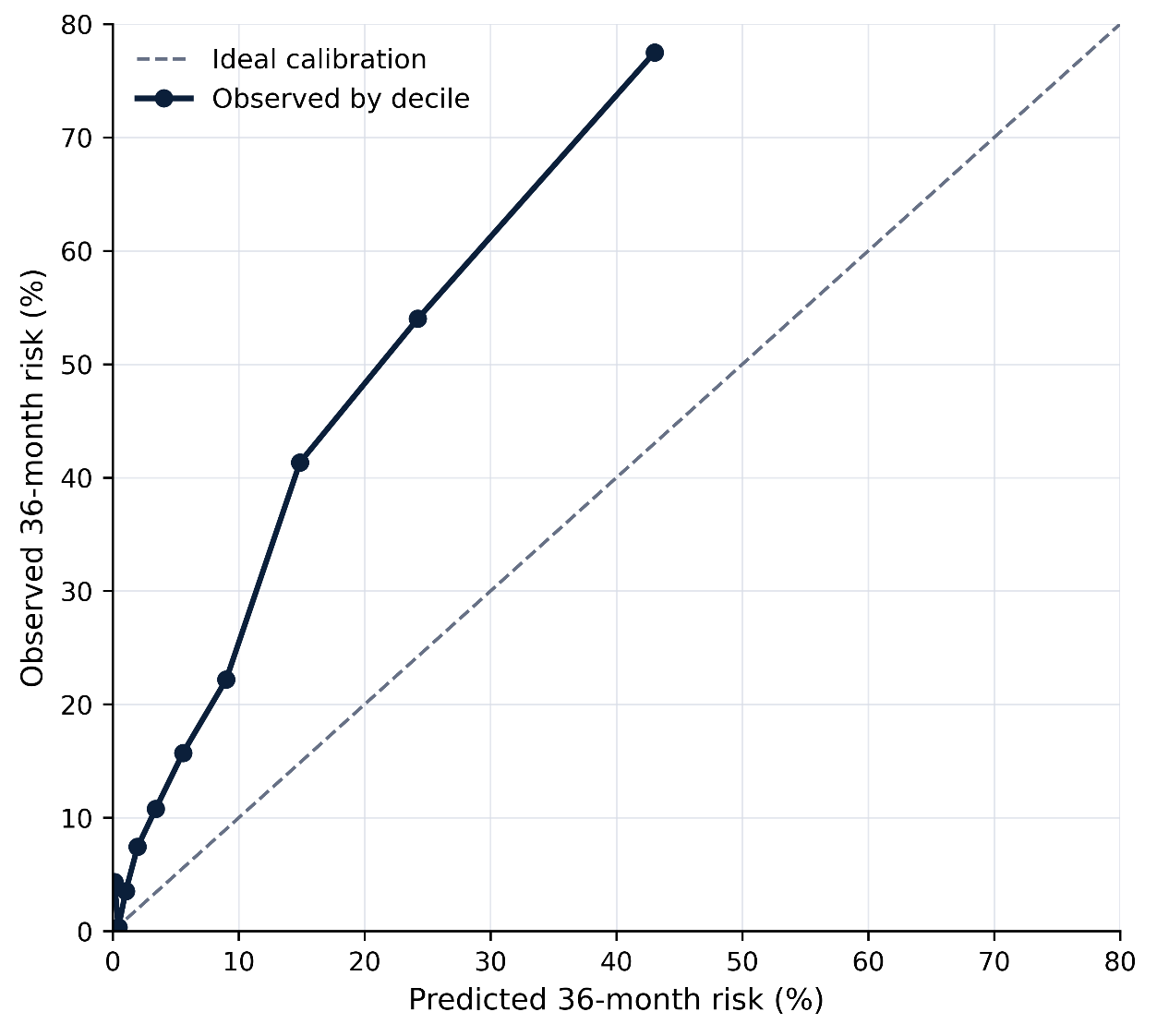


Figure S16. Approximate diagnosis of proportional hazards.


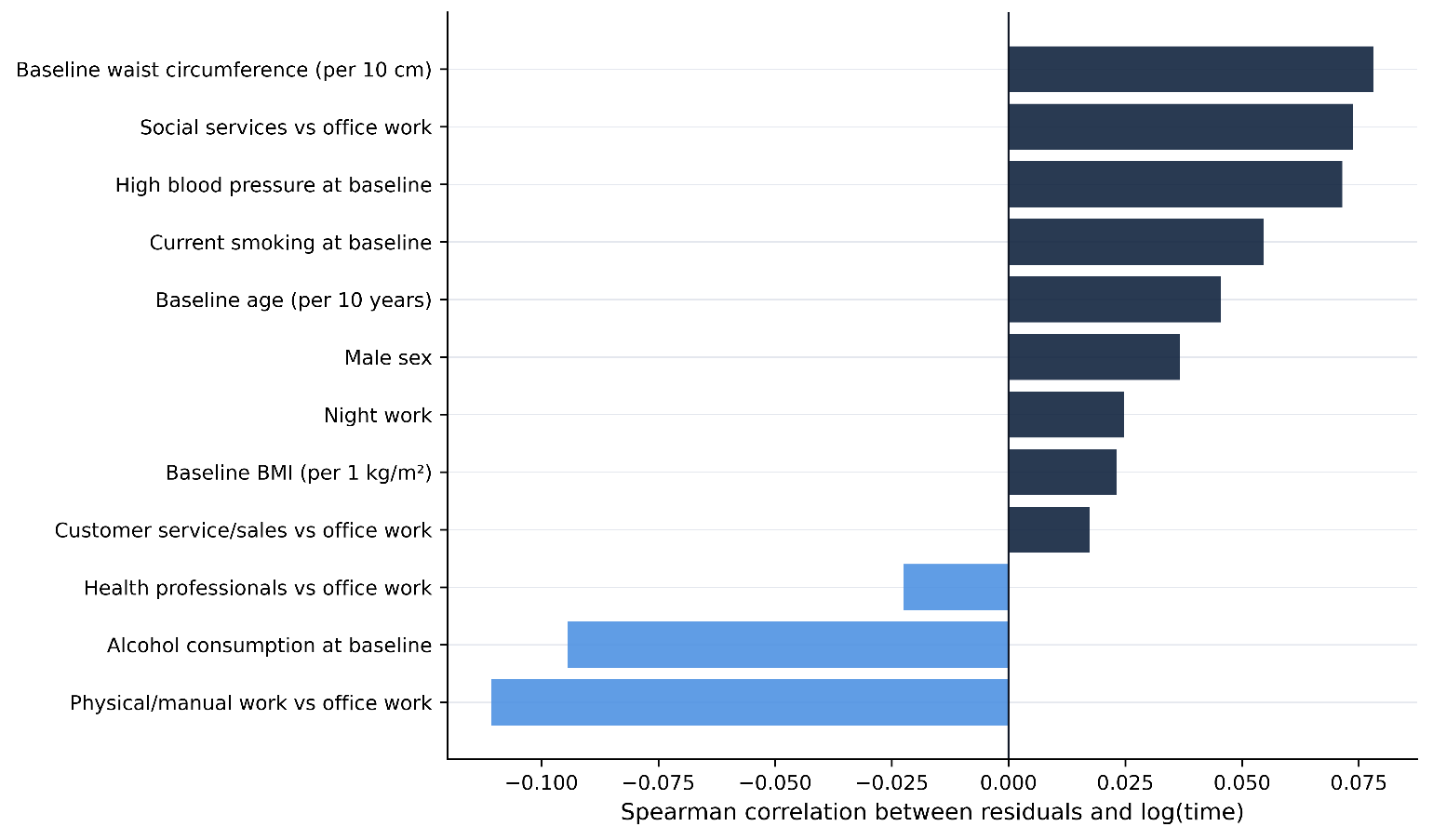


Figure S17. C-index of the final model by subgroup.


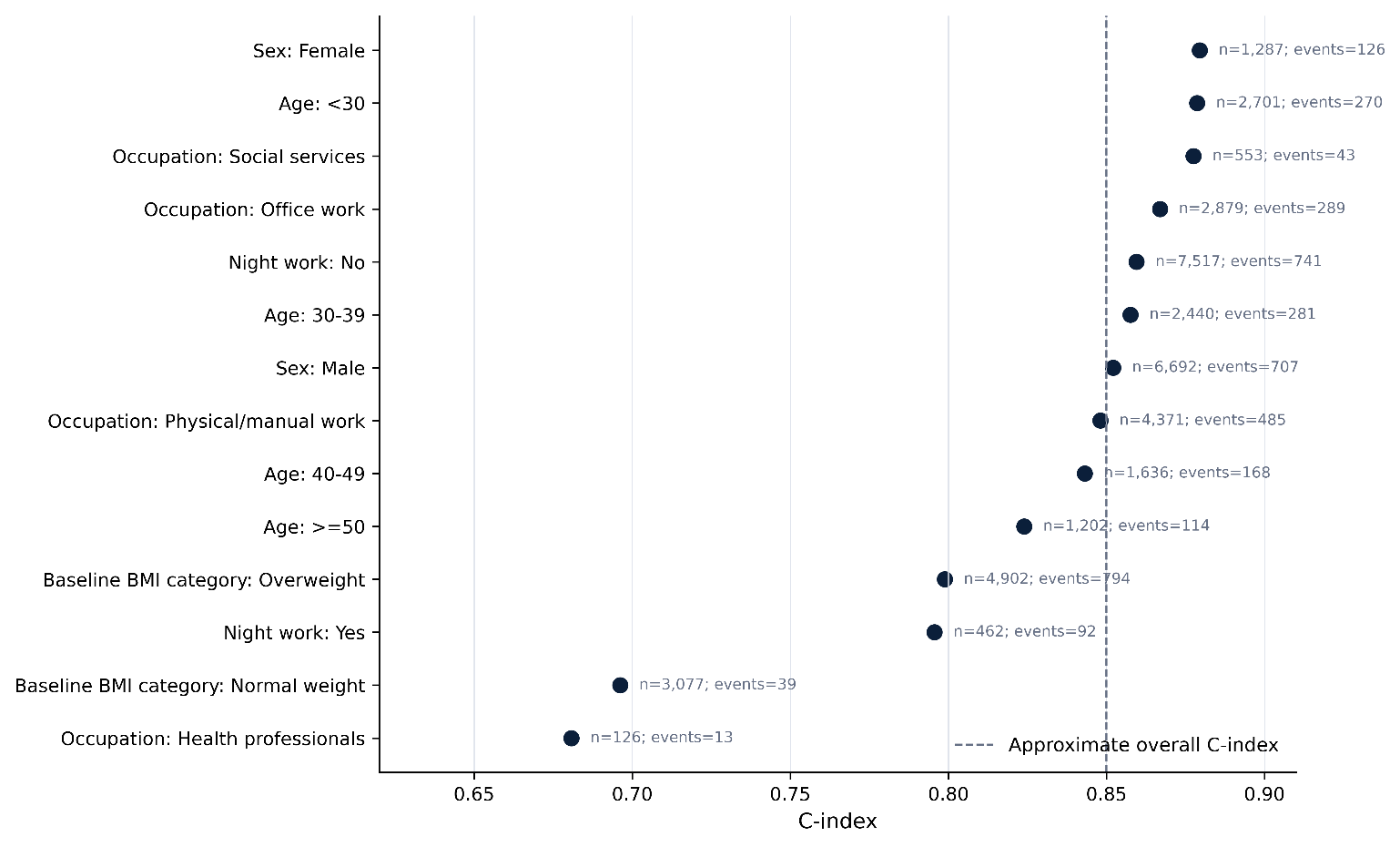


Figure S18. Summary of the exploratory landmark analysis.


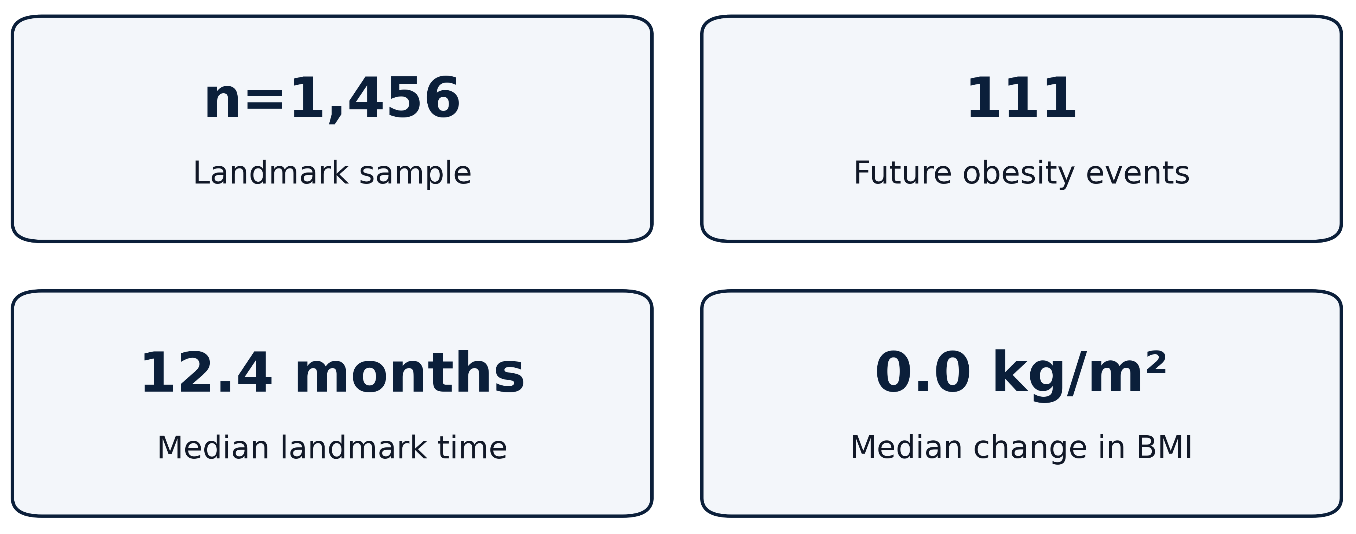

Supplement: Multimedia component 1 [file mmc1.docx]
